# Functionalization strategies of polymeric nanoparticles for drug delivery in Alzheimer’s disease: Current trends and future perspectives

**DOI:** 10.3389/fnins.2022.939855

**Published:** 2022-08-04

**Authors:** Livia La Barbera, Emanuele Mauri, Marcello D’Amelio, Manuele Gori

**Affiliations:** ^1^Department of Medicine and Surgery, Università Campus Bio-Medico di Roma, Rome, Italy; ^2^Santa Lucia Foundation, IRCSS, Rome, Italy; ^3^Department of Engineering, Università Campus Bio-Medico di Roma, Rome, Italy; ^4^Institute of Biochemistry and Cell Biology (IBBC) - National Research Council (CNR), Rome, Italy

**Keywords:** Alzheimer’s disease, neurodegeneration, blood-brain barrier, polymeric nanoparticles, nanotheranostics, drug delivery, amyloid-β, nanomedicine

## Abstract

Alzheimer’s disease (AD), the most common form of dementia, is a progressive and multifactorial neurodegenerative disorder whose primary causes are mostly unknown. Due to the increase in life expectancy of world population, including developing countries, AD, whose incidence rises dramatically with age, is at the forefront among neurodegenerative diseases. Moreover, a definitive cure is not yet within reach, imposing substantial medical and public health burdens at every latitude. Therefore, the effort to devise novel and effective therapeutic strategies is still of paramount importance. Genetic, functional, structural and biochemical studies all indicate that new and efficacious drug delivery strategies interfere at different levels with various cellular and molecular targets. Over the last few decades, therapeutic development of nanomedicine at preclinical stage has shown to progress at a fast pace, thus paving the way for its potential impact on human health in improving prevention, diagnosis, and treatment of age-related neurodegenerative disorders, including AD. Clinical translation of nano-based therapeutics, despite current limitations, may present important advantages and innovation to be exploited in the neuroscience field as well. In this state-of-the-art review article, we present the most promising applications of polymeric nanoparticle-mediated drug delivery for bypassing the blood-brain barrier of AD preclinical models and boost pharmacological safety and efficacy. In particular, novel strategic chemical functionalization of polymeric nanocarriers that could be successfully employed for treating AD are thoroughly described. Emphasis is also placed on nanotheranostics as both potential therapeutic and diagnostic tool for targeted treatments. Our review highlights the emerging role of nanomedicine in the management of AD, providing the readers with an overview of the nanostrategies currently available to develop future therapeutic applications against this chronic neurodegenerative disease.

## Introduction

Alzheimer’s disease (AD) is the most common cause of dementia and the fifth leading cause of death in adults older than 65 years only in the United States (Centers for Disease Control and Prevention [CDC]). At present, the world societal and economic burden of neurodegenerative diseases, in particular AD, is an enormous health care problem. In the United States, total costs for AD care, between direct and indirect, are estimated to increase to more than $1 trillion by 2050 together with the number of new patients as the population ages (Deb et al., 2017; Farro et al., 2020). Despite this major public health scourge, the search for a successful cure remains a daunting challenge.

Nanotechnologies applied to the field of neuroscience will undoubtedly help advance our ability to diagnose and treat several neurodegenerative diseases, including AD, in forthcoming time. In particular, the new field of theranostics nanomedicine, which enables at the same time diagnosis and targeted drug delivery, has shown significant results in the dawning era of personalized medicine. As a matter of fact, achieving early diagnosis would enable improved disease outcomes.

Alzheimer’s disease treatment using new biologic drugs, such as recombinant proteins or gene therapies, needs their reengineering and chemical modification to allow these molecule therapeutics to cross the blood-brain barrier (BBB) from circulating blood (Pardridge, 2020). The BBB has the function of blocking most of the large and polar molecules, including medications, from entering the central nervous system (CNS) with very high efficiency. The BBB is indeed permeable only to ions (e.g., O_2_, CO_2_) and small lipid-soluble molecules (less than 400 Dalton) (Banks, 2009). According to the Comprehensive Medicinal Chemistry database, only 5% of over 7,000 drugs are suitable for entering the CNS, ensuring sufficient penetration (Meng et al., 2019).

Since traditional drug delivery systems generally fail to cross the BBB, they result inefficient in disease prevention and pharmacological treatment. Among several examples, specific monoclonal antibodies (MAb) harnessed in AD immunotherapy, metal chelators or natural plant-derived compounds that possess interesting features as potential therapeutics against AD, in a free form do not reach the brain at desired therapeutic amounts because of their own physical and chemical characteristics, such as hydrophilic nature, large size, very low half-life and limited bioavailability due to their rapid metabolism, hence leading to subtherapeutic levels in humans (Andersen, 2004; González-Santiago et al., 2010; Sun et al., 2016). Conversely, nanoparticles (NPs), due to their small size (usually ranging from 1 to 100 nm), improved pharmacokinetics and high stability in biological environments, surface-engineered adhesive properties through the conjugation with chemical groups and moieties along with surface positive charges that favor the interaction with the negatively charged surface of the BBB endothelial cells, represent an invaluable tool to enhance cellular uptake in the brain and drug absorption (Jallouli et al., 2007), thus being able to easily penetrate the BBB and reach the desired brain area protecting the carried drug (Vert et al., 2012; Faiyaz et al., 2021). Moreover, because of their high safety profile and biocompatibility, combined with the possibility to be tailored by chemical modifications to confer them the desired features, NPs have captured the attention of the biomedical community (Swierczewska et al., 2016; Bassas-Galia et al., 2017; Patra et al., 2018). Finally, systemic toxicity is a harmful side effect that must be considered when traditional drug administration methods are employed to take therapeutics to the brain. This limitation can be significantly overcome using nanoscopic particles for more controlled and gradual release of therapeutic compounds, thereby limiting their potential noxious side effects.

In the last few decades nanomedicine achieved outstanding preclinical breakthroughs in the diagnosis and treatment of neurodegenerative disorders including, among the others, Parkinson’s disease, amyotrophic lateral sclerosis, Huntington’s disease and Alzheimer’s disease (Kabanov and Gendelman, 2007; Kumar et al., 2020; Mukherjee et al., 2020). Given the paucity of definitive therapeutic treatments and the lack of resolutive cures against AD, nanomedicine and nanoparticle-based theranostics deserve further investigation in such a context, with the ambition to move toward a clinical success.

Remarkable works have partially explored the wide range of possibilities offered by these nanotechnological approaches to provide early and non-invasive diagnostic tools, as well as potential targeted therapeutic strategies against several neurological diseases, moving from bench to bedside (Kumar et al., 2020). To date, the broad spectrum of nanotechnology-based materials and approaches includes: exosomes, hydrogels, dendrimers, quantum dots (QDs), carbon nanotubes, nanodiscs, nanowires, nanostructured sensors, metal- and alloy-based NPs, lipid-based NPs, inorganic and organic NPs. However, herein we will focus on polymeric NPs that currently represent one of the most innovative, versatile and non-invasive tools for brain-targeted drug delivery. Hence, in this review article we will summarize the most recent advances in the application of brain-targeted polymeric nanoparticles to the treatment of neurodegenerative diseases, with a specific focus on AD. We will also thoroughly discuss key challenges, pitfalls and future opportunities associated with this emerging and promising technology.

## Alzheimer’s disease

**FIGURE 1:**
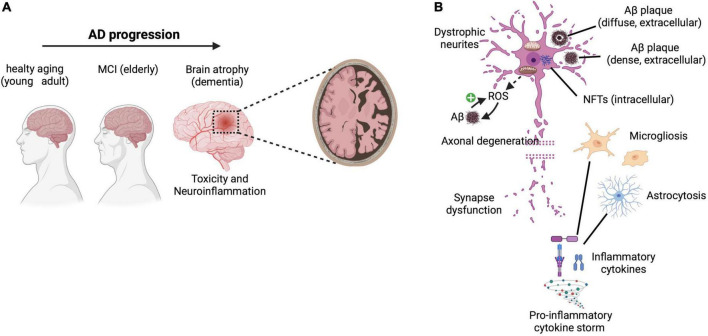
AD features and hallmarks. **(A)** Progression of AD from (i) healthy aging in young adults through the appearance of (ii) mild-cognitive-impairment (MCI) in the elderly, toward (iii) clinical AD encompassing dementia, loss of memory and cognitive functions due to brain atrophy (mainly characterized by neurotoxicity and inflammation). **(B)** The main neuropathological hallmarks include lesions, such as diffuse and dense extracellular amyloid-β (Aβ) plaques, intracellular neurofibrillary tangles (NFTs), glial activation and responses (i.e., microgliosis and astrocytosis), release of pro-inflammatory cytokines and mediators as well as oxidative stress (with generation of ROS associated with Aβ accumulation that in turn may further promote additional ROS production in a positive feedback mechanism), which altogether lead to dystrophic neurites, axonal degeneration, and synapse dysfunction associated with severe neurological impairments. Reactive oxygen species (ROS). Created with BioRender.com.

Alzheimer’s disease is a progressive and irreversible neurodegenerative disorder, principally characterized by loss of memory and cognition (D’Amelio and Rossini, 2012). The trajectory of disease begins from healthy aging, mild-cognitive-impairment (MCI) and proceeds toward clinical AD with dementia. However, more than 50% of individuals develop non-cognitive, neuropsychiatric symptoms and personality changes (Lyketsos et al., 2011). Indeed, these symptoms, particularly apathy, depression, aggression, personality changes and circadian rhythm disturbances are subtly observed very early in AD (Lyketsos et al., 2011; Masters et al., 2015; Ismail et al., 2016; Alves et al., 2017; Musiek et al., 2018) and persist in parallel with the different stages of deterioration of cognitive function (summarized in Figure 1A).

At histopathological level, the main AD hallmarks are: (i) extracellular β-amyloid (Aβ) deposits, and (ii) intracellular neurofibrillary tangles (NFTs), containing bundles of paired helical filaments of the microtubule-associated hyperphosphorylated protein tau (Grundke-Iqbal et al., 1986; Nukina and Ihara, 1986). These aggregates cause toxicity and lead to synapse dysfuntions, inflammation, oxidative stress and, ultimately, to neuronal loss (Figure 1B; Congdon and Sigurdsson, 2018).

Alzheimer’s disease is a complicated and multifactorial disorder, and many hypotheses have been developed, including Aβ (Selkoe and Hardy, 2016), tau (Arnsten et al., 2021), cholinergic (Terry and Buccafusco, 2003; Chen and Mobley, 2019), and dopaminergic degeneration (Nobili et al., 2017; Cordella et al., 2018; De Marco and Venneri, 2018; Serra et al., 2018, 2021; Bozzali et al., 2019; Iaccarino et al., 2020; Venneri and De Marco, 2020; La Barbera et al., 2021; Sala et al., 2021; Spoleti et al., 2022), oxidative stress (Markesbery, 1997), inflammation (Kinney et al., 2018), etc. How much further we have to go before deciphering all the molecular mechanisms underlying the onset and evolution of AD and, in turn, the putative druggable targets of the disease is not yet known, however, many studies have been done to find a potential therapeutic strategy.

In the next paragraphs, we will describe briefly the most accredited AD hypotheses for identifying possible therapeutic targets.

### Aβ hypothesis

In the last 20 years, many works either *in vitro* or using genetically modified animal models have agreed that Aβ somehow contributes to the progression of AD, making it a prime target for various therapeutic interventions. Aβ is a 4.2 kDa peptide normally consisting of a heterogeneous group of peptides of varying length (i.e., between 38 and 43 amino acids) (Golde et al., 2000; Selkoe, 2001), formed by sequential cleavages of the amyloid precursor protein (APP), by β- and γ-secretase (Zheng and Koo, 2011; Makin, 2018). The principal genetic causes of AD are mutations in different genes, such as APP (Goate et al., 1991), γ-secretase proteins presenilin-1 and presenilin-2 (PSEN1-2) (Scheuner et al., 1996), which result in the production of aggregation-prone Aβ peptides, called oligomers. Functionally, Aβ oligomers can interact with a wide variety of transmembrane receptors, leading to the activation of different neurotoxic pathways, among which endoplasmic reticulum (ER) stress response, mitochondrial dysfunction, tau tangle formation, DNA damage and inflammatory response (Goate et al., 1991; Hardy and Allsop, 1991; Hardy and Higgins, 1992; Cavallucci et al., 2012; Forner et al., 2017). According to these mechanisms, since elevated levels of Aβ may likely underlie its pathogenicity, treatment strategies are focused either on targeting the excessive generation of Aβ, due to alterations in β- and γ-secretase (Vassar and Citron, 2000; Cummings et al., 2016) or on its faulty clearance mechanisms, as alterations in the process of autophagy (La Barbera et al., 2021; Nobili et al., 2021). Unfortunately, targeting of γ-secretase produced some undesirable side effects, because of its physiological role in the cleavage of multiple transmembrane protein substrates (Periz and Fortini, 2004), and its control over many biological processes, such as cell differentiation, proliferation, and/or survival. Similarly, β-secretase has different important substrates (Klaver et al., 2010) and inhibiting its activity could have toxic consequences.

### Tau hypothesis

The other important hallmark of AD are the pathological NFTs, composed of phosphorylated tau protein, located both in cell body and dendrites or axons. Tau is a microtubule-associated protein that stabilizes microtubules and is frequently phosphorylated. The hyperphosphorylation of Tau, but also other abnormal post-translational modifications, or proteolytic cleavage (Wischik et al., 1988; Goedert et al., 1992; Novak et al., 1993; Liu et al., 2004; Guillozet-Bongaarts et al., 2005; Min et al., 2010; Kolarova et al., 2012; Flores-Rodríguez et al., 2015), render the protein more aggregation-prone and reduce the affinity with microtubules, inducing its dissociation. This aberrant interaction has negative effects on neuronal physiology, causing disintegration of cytoskeletal system, collapse of neuronal transport, alterations signaling system and mitochondrial integrity, and resulting in neuronal damage, synaptic impairment and cell loss at last (Iqbal et al., 2010). After numerous failures of the therapeutic strategies acting against Aβ, and the different works that strongly associated tau alterations to AD, many researchers focused their attention also on therapeutic strategies to target tau (Panza et al., 2016) by acting on the block or prevention of tau phosphorylation, through the activity of specific kinases or phosphatases, as well as on either inhibiting its aggregation or promoting its clearance (Iqbal et al., 2014; Anand and Sabbagh, 2015; Pedersen and Sigurdsson, 2015).

### Oxidative stress hypothesis

Another important player in AD pathogenesis is the oxidative stress, caused by an imbalance between production of reactive oxygen species (ROS) and antioxidant defense. Specifically, neurons are post-mitotic cells that have a high metabolism and need high levels of oxygen that undergoes mitochondrial respiration, increasing ROS production. In a normal condition, ROS are kept at low levels by antioxidant molecules, such as glutathione and vitamins, but during aging these antioxidant defense mechanisms decrease their activity. For example, glutathione is a ROS scavenger and its concentration was observed to be reduced both in experimental models (Chen et al., 1989; Iantomasi et al., 1993; Sasaki et al., 2001; Liu, 2002; Wang et al., 2003) and in AD patients (Gsell et al., 1995; Zhu et al., 2006; Chen et al., 2022). In this way, the rate of ROS production exceeds the antioxidant ability of ROS buffering, leading to increased oxidative stress. This condition manifests in the brains as presence of oxidized proteins, lipid peroxidation, glycoxidation, formation of toxic species (i.e., free carbonyls, ketones, peroxides, etc.) as well as nuclear and mitochondrial DNA oxidative modifications (Cini and Moretti, 1995; Forster et al., 1996; Schippling et al., 2000; Ramassamy et al., 2001; Bassett and Montine, 2003; Abd El Mohsen et al., 2005; Lovell and Markesbery, 2007). Moreover, oxidative stress is associated with Aβ accumulation, and Aβ in turn increases oxidative stress (Figure 1B; Butterfield and Lauderback, 2002; Butterfield et al., 2002, 2007; Gibson et al., 2004; Mohmmad Abdul et al., 2006; Sultana and Butterfield, 2008; Cheignon et al., 2018). All this evidence highlights the importance of an antioxidant therapeutic strategy to reduce oxidative stress and Aβ toxicity. However, antioxidant treatment with vitamin C and E results in no significant clinical protective effects (Masaki et al., 2000; Kontush and Schekatolina, 2004; Boothby and Doering, 2005; Kryscio et al., 2017), suggesting that antioxidant defense alone is not able to induce neuroprotective effects, but it is necessary a combination of more than one factor.

### Inflammation hypothesis

Neuroinflammation is another typical hallmark of AD (Leng and Edison, 2021), and the relationship between microglia and astrocyte activation and the appearance of other histological AD-related alterations is unclear and heavily debated, with conflicts regarding their protective or detrimental contribution (Fiala et al., 2007; Keren-Shaul et al., 2017). However, the current hypothesis is that activation of glia may act as a neuroprotective mechanism for Aβ phagocytosis at early disease stages but become detrimental later on. Specifically, since the early stages of the disease, AD brains are characterized by the presence of reactive microglia and astrocytes, which at the beginning release anti-inflammatory factors in the microenvironment, and later neurotoxic pro-inflammatory mediators, thereby exacerbating neurodegeneration. In this context, it is still debated whether inflammation directly causes neurodegeneration or the neuronal loss induces glia activation, generating a positive feedback loop in which inflammatory cytokines and neurotoxic molecules released by activated glial cells promote both neurodegeneration and self-perpetuating inflammatory response (Figure 1B). Anyway, many non-steroid anti-inflammatory drugs failed to produce interesting benefits in clinics. This because it is necessary a time-specific intervention for inducing beneficial and not detrimental effects, due to the different functions of specific microglia (McGeer and McGeer, 2015; Bolós et al., 2017; Chen et al., 2017; Hirbec et al., 2017). In fact, recent research has been focused on the conversion of pro-inflammatory microglia to anti-inflammatory microglia, by increasing its ability to phagocyte Aβ, and attenuating extracellular Aβ release and aggregation (McGeer and McGeer, 2015; Guo et al., 2022).

## Therapeutic strategies for Alzheimer’s disease treatment

Many therapeutic strategies were identified for the treatment of AD, but only few drugs were approved by the FDA for the improvement of AD symptoms. These drugs are principally cholinesterase inhibitors (ChEIs) that block the hydrolysis of acetylcholine (Rogers and Friedhoff, 1996; Bryson and Benfield, 1997; Woodruff-Pak et al., 2007; Cutuli et al., 2013), or N-methyl-D-aspartate (NMDA) receptor blockers (Linkins et al., 2000; Riepe et al., 2006), which act reducing glutamate activity.

The first class of chemicals (ChEIs, such as Galantamine, Rivastigmine, and Donepezil) has long been considered in the US as the best choice for treating mild to moderate AD cases showing improvements in cognitive function (Birks, 2006; Thoe et al., 2021).

A representative example of their use, combined with a nanoparticulate drug delivery system, was provided for potential brain targeting in AD (Bhavna et al., 2014). In this work, Donepezil-loaded poly lactic-co-glycolic acid (PLGA) NPs showed sustained release and increased uptake in rat brain by *in vivo* studies using gamma scintigraphy techniques. The first generation of ChEI approved by the FDA, namely Tacrine hydrochloride (THA), showed, however, serious side effects and poor patient compliance in long-term therapy (Madden et al., 1995) and a short half-life with high elimination rate in pharmacokinetic studies (Forsyth et al., 1989). Therefore, these limitations made it necessary the use of polymeric NPs as more effective THA delivery carriers compared with free drugs, including Methoxypoly (ethylene glycol)-Polycaprolactone (mPEG-PCL) NPs in rat brain (Sathesh Kumar and Felix Joe, 2017) or poly(n-butyl cyanoacrylate) (PBCA) NPs coated with polysorbate 80 (aka Tween 80, T80) in mouse brain (Wilson et al., 2008b). Alternatively, different formulations of lipid NPs loaded with Tacrine or conjugating Tacrine with model amphipathic peptide (MAP) showed high encapsulation efficiencies, low cytotoxicity in the neuroblastoma cell line SH-SY5Y and thus improved therapeutic efficacy as potential drug delivery systems (Silva et al., 2020). However, these therapies with ChEIs and NMDA are only symptomatic and not curative or preventative.

In the last two decades, researchers have developed a lot of small molecule drugs focused on reducing Aβ oligomers and NFTs. However, only 2% of all drugs were active in the CNS, due to the inability to cross the BBB (Lipinski, 2000; Pardridge, 2005). For example, homotaurine (tramiprosate) is a small molecule that acts by inhibiting the formation of Aβ dimers. This molecule failed in clinical trials because of its limited transport across the BBB resulting in no changes in cognitive functions (Aisen et al., 2011). There are many other small molecules that act against Aβ, reducing soluble oligomers; one of these drugs is cromolyn, which is now in clinical trials for AD (NCT04570644), although it has properties that suggest a minimal transport through the BBB. Despite all the resources used so far, no small molecules have been approved from the FDA since 2003 (Sun and Benet, 2020), because of their minimal BBB penetration.

In recent times, AD research has focused on immunotherapy using MAb as alternative approach, directly targeting Aβ and leading to its clearance (Bayer et al., 2005; Gilman et al., 2005; Holmes et al., 2008; Salloway et al., 2009; Laskowitz and Kolls, 2010; La Porte et al., 2012; Burstein et al., 2013; Landen et al., 2013; Bouter et al., 2015; Gandy and Sano, 2015; Carlson et al., 2016; Ultsch et al., 2016; The Lancet Neurology, 2017). Even if these strategies elicited positive effects in reducing Aβ charge and amyloid plaques from the brain, such clearance mechanism is not able to prevent progressive neurodegeneration (Holmes et al., 2008). Moreover, immunotherapy-based strategies failed in clinical trials because of different side effects (Bayer et al., 2005; Gilman et al., 2005). For example, preclinical mouse models of AD showed that intra-cerebral injection of anti-Aβ-antibody (AAA) resulted in reduction of Aβ plaques (Solomon et al., 1997), whereas its systemic administration failed, because of the difficulty to penetrate the BBB (Boado et al., 2007). Importantly, although the usage of higher doses of AAA promoted the brain entry, it had undesirable side effects, as cerebral microhemorrhage (Wilcock et al., 2004).

The first AAA to enter in clinical trials for AD was bapineuzumab (Rinne et al., 2010), which was shown to penetrate mouse brain in only 0.07% of injected dose when administer intravenously (Bard et al., 2012). Similar to observations in mouse models, administration of higher doses of bapineuzumab in patients, caused side effects as vasogenic edema and BBB disruption (Sperling et al., 2012), resulting in clinical trial failure.

Despite the negative results observed, since 2005 to date, more than a dozen of AAAs entered in clinical trials, but most of these trials terminated unsuccessfully and with no FDA approval. One exception is RO7126209, deriving from the fusion between an AAA that failed in clinical trials (Lozupone et al., 2020), and an antibody (Ab) against transferrin receptor (Niewoehner et al., 2014), which mediated the BBB transport (Pardridge, 2020). The clinical trial for this AAA is still running (i.e., phase 1 completed). Aducanumab, a MAb recently approved by the FDA, seems to be confined to the brain blood volume (Pardridge, 2019), but it can reduce Aβ plaques in AD (Sevigny et al., 2016), probably due to BBB disruption. However, a clinical trial with aducanumab failed to reduce cognitive deficits (Pardridge, 2019). Indeed, solanezumab, another humanized monoclonal IgG1 Ab acting against the mid-region of Aβ, failed in clinical trial (Doody et al., 2014) but seems to have some cognitive benefits in the MCI stage of the disease (Panza et al., 2014). On this basis, Aβ was considered one of the first direct NP target for the delivery of drugs, such as AAAs. Similar to AAAs, anti-tau antibodies (ATAs) were designed for recognizing and binding hyperphosphorylated-tau. Some ATAs are in phase I and II clinical trials (Li and Götz, 2017; Novak et al., 2017; Sopko et al., 2020), but most failed in phase III (Li and Götz, 2017). In fact, similar to Aβ-targeted immunotherapy, most of the ATAs are not able to cross an intact BBB (Janowicz et al., 2019), and led to toxic side effects, such as vascular edema, encephalomyelitis associated with neurological deficits, axonal damage and inflammation (Rosenmann et al., 2006; Sperling et al., 2012). Specifically, polymeric NPs can be tailored and harnessed to target specific brain molecular structures and components, including nucleic acids, cellular membrane, proteins, peptides as well as NFTs by using ATAs. In spite of the various ongoing phases of clinical trials that use several Aβ-specific Abs and the high expectations placed in the use of immunotherapy, very few studies have combined together Abs and NPs as therapeutic AD regimen. The first work that used Aβ_1–42_ monoclonal antibody-tagged polymeric NPs was carried out in the Tg2576 mouse model of AD. In this study it was observed not only a brain reduction of the soluble Aβ peptide but also a total recovery in memory function in the treated AD mice (Carradori et al., 2018).

Related to the obstacle of crossing the BBB, any drug therapeutic efficacy depends on the administered intracellular dosage that effectively reaches the desired brain area. For these reason, multidisciplinary strategies based on the combination between specific therapeutics and nanocarriers with targeting motifs that can bind to molecules expressed on the cell of interest have led to the development of nanoformulates capable to outperform the conventional administration routes. In particular, polymeric nanoparticles exhibit specific advantages compared to inorganic-based nanosystems related to the opportunity to tune their physicochemical properties depending on the specific applications. The wide selection of starting materials (i.e., monomers or preformed polymers) and the synthesis protocols enable the modulation of charge, size, shape, surface area, encapsulation efficiency and controlled drug delivery performances. Notably, size-dependent transport was observed in animal models where smaller or functionalized NPs resulted in a higher accumulation and release of the payload in the targeted area (Cruz et al., 2016). Additionally, key parameters are related to the NP stiffness and shape, and to the ligand density. Crosslinking density and polymer chemical structures can modulate the stiffness of the final nanocarrier affecting the BBB crossing efficiency and intracellular accumulation of the cargo: as an example, Anselmo et al. (2015) had monitored the internalization of Poly(ethylene glycol) diacrylate (PEGDA) NPs characterized by a stiffness ranging from 10 to 3,000 kPa showing a higher uptake for softer NPs in the first hours; whereas, more recently (Zheng et al., 2021) demonstrated that lower elasticity of PEG-block-poly(pentafluorophenyl methacrylate) NPs led to increased transcytosis and brain accumulation, despite shorter blood circulation times.

Regarding the NP functionalization with ligands, high density of these motifs on NP surfaces may generate undesired side effects, such as size increase, steric hindrance, and decreased stealth behavior to the immune system *in vivo* (Alkilany et al., 2019). As a result, controlling cellular uptake of NPs in relation to ligand density has gained increasing interest in the nanomedicine field, in particular regarding the choice of the most appropriate linker molecule involved in the polymer-ligand conjugation: latest researches have revealed that higher NP internalization occurs with shorter linker length (Abstiens et al., 2019). Regulation of NP avidity, intended as enough ligand density along with sufficient ligand–receptor binding affinity, can be addressed using antibody-decorated polymeric NPs with the aim to improve the delivery into the brain parenchyma and avoid the agglomeration in the microvasculature (Deng et al., 2019).

Additionally, the rational choice of the crosslinking chemistry, both for the design of the NPs network and for their functionalization, can affect the degradation behavior of the nanoscaffolds, minimizing the risk of toxicity or other side effects associated to a considerable intracellular accumulation, therefore: linkers cleavable under hydrolysis, thermolysis or enzymatic activity represent suitable alternatives to improve the degradability of the NPs in the intracellular environment. Nevertheless, this type of crosslinking could reduce the stability of the nanocarriers and, as a result, the controlled drug release. For these reasons, depending on the final target, NPs are generally designed considering a balance between high stability vs. slow degradability over time (Wen et al., 2017). NPs for AD treatment meet the biocompatibility criteria thanks to the smart combination of both natural and synthetic polymers, estimating the optimal composition *via* preliminary *in vitro* assays for cell viability and proliferation (e.g., MTT, MTS, Live/Dead, BrdU assay) (Voigt et al., 2014).

Overall, the ability to address NP synthesis toward these specific needs through the smart conjugation of polymers and biological/chemical motifs paves the way to these nanomaterials for very promising therapeutic and clinical approaches (Zhang et al., 2021a). Nowadays, NPs are being extensively used in medicine because of their high sensitivity in molecular recognition and detection, and for the absence of side effects. Importantly, it is worth noting that NPs have the ability to easily cross the BBB, one of the most significant hurdles for an effective delivery of drugs to the brain, which may limit their therapeutic effect.

## The blood-brain barrier obstacle

**FIGURE 2:**
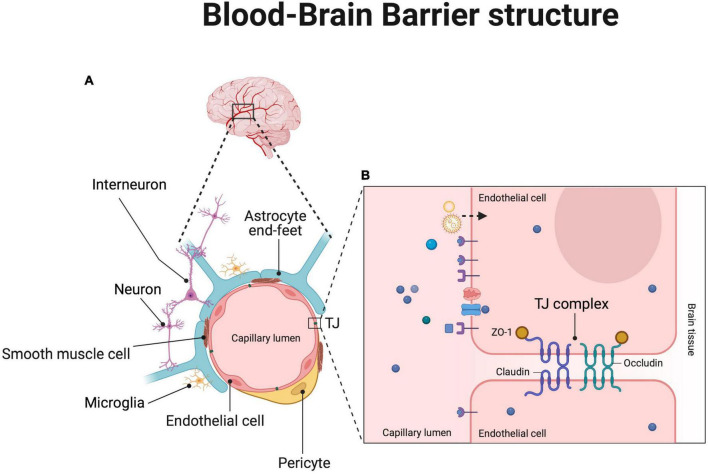
Schematic representation of the BBB structure and possible drug delivery pathways. **(A)** Blood vessels of the human brain and, down below, magnified square inset of a vessel cross section showing the different cell components that surround and ensheath the endothelium surface made of a monolayer of endothelial cells sealed by tight junction (TJ) complexes (enlarged in panel B). In the enlarged box in **(B)** are schematized passive (dashed black arrow and micelles or lipid NPs) and active (violet receptors as well as pink and blue transporters and channels through the endothelial cell membrane) transport mechanisms of different molecules, ligands and NPs (depicted with different colors) across the endothelial cells of the BBB mediated by surface receptors, channels and transporters as further detailed in Figure 3. Created with BioRender.com.

The BBB is a selective semipermeable interface between neuronal tissue and blood vessels that form the neurovascular unit, with the main role of helping normal brain homeostasis, regulating influx and efflux transport, and ensure protection, mediating the communication between CNS and peripheral one (Rhea and Banks, 2019). The BBB represents a dynamic boundary that impedes the entry of blood borne pathogens, toxic plasma components, blood cells and toxic chemicals into the brain, allowing at the same time selective passive transport of nutrients and metabolites (Obermeier et al., 2013). Owing to its nature as a selective filter, the BBB prevents effective doses of large and hydrophilic drugs reaching the site of interest into the brain. Therefore, using nano-sized NPs can help overcome this physiological hurdle facilitating drug delivery in a smoother manner. The BBB represents a dynamic boundary with a dishomogeneous composition throughout the brain, with diverse types of blood vessels, including arterioles, capillaries and venules. It is made up of the following cell types: brain endothelial cells, pericytes, astrocytes and associated neurons in the near proximity of the BBB (Figure 2), as further reviewed in Rhea and Banks (2019) and Sweeney et al. (2019). The combination of the vascular components [i.e., endothelium, pericytes and smooth muscle cells (SMCs)], glial cells (i.e., astrocytes and microglia) and neuronal cells creates an interacting compartment called neurovascular unit (NVU), which is essential for regulating the BBB permeability, neurovascular coupling, cell-matrix interactions and in turn for contributing to the whole tissue homeostasis, including angiogenesis and neurogenesis (Sweeney et al., 2019).

### Endothelial cells

The main physical barrier of the BBB is represented by a monolayer of endothelial cells of the CNS that depends on continuous intercellular complexes of tight junctions (TJs) and adherens junctions (AJs) for its integrity (Figure 2B; Armulik et al., 2010). These TJ and AJ complexes increase the electrical resistance and prevent paracellular diffusion of molecules, thereby representing an interesting target also for nano-based drug delivery strategies to cross the AD BBB (Tietz and Engelhardt, 2015). This endothelial compartment of the BBB has the peculiar feature to be supplied with plenty of TJ complexes between cells of the brain vessels, which are made of different proteins; these include: claudins, occludin and junction adhesion molecules that are all in close relation with the membrane-associated guanylate-kinase zonula occludens family of scaffolding proteins (i.e., ZO-1, ZO-2, ZO-3) that link TJs to the actin cytoskeleton (Figure 2B; Dadas et al., 2019; Mukherjee et al., 2020). Endothelial cells create therefore a stronger barrier with a large surface area, limited vesicle-mediated transcellular transport and low pinocytosis (Reese and Karnovsky, 1967). Albeit this tight barrier, endothelial cells are polarized and possess a complex cellular machinery of molecular transporters that allows proteins and other molecules to cross the brain cortex in both directions. For instance, it is known that specific receptors are highly expressed on the luminal side of endothelial cells across the BBB, such as the transferrin receptor (TfR) on brain capillary endothelial cells that is a potential target for developing effective drug delivery solutions (Bourassa et al., 2019). These receptors mediate endocytosis and transcytosis of various molecules, such as transferrin, hormones and neuropeptides, to help them cross the BBB and could therefore be exploited, as usually done for the passage of chemotherapeutic agents, for enabling surface-modified NPs to overcome the BBB (Saraiva et al., 2016). In addition to the above mechanism, adsorptive-mediated transcytosis (AMT) is the principal way used for macromolecular transport, which is based on electrostatic interactions between cationic molecules and anionic microdomains present on the membrane of the brain capillary endothelial cells (Lu, 2012). These and other surface molecules and domains may therefore represent appealing “entry routes” expressed by the endothelial compartment for devising smart strategies of NP-mediated BBB penetration in future works.

### Pericytes

In the human brain, the main contribution to the cerebral blood vessels is given by capillaries, along which pericytes maintain the BBB integrity and function. Pericytes, which lie on the outer surface of endothelial cells, share indeed a basement membrane with endothelial cells of brain capillaries (Figure 2A; Armulik et al., 2010; Bell et al., 2010; Sweeney et al., 2016), and interact with these cells through N-cadherins (Gerhardt et al., 2000). The intercellular communication between endothelial cells and pericytes is ensured by Gap junction CX43 hemichannels (Winkler et al., 2011; Sweeney et al., 2016), peg and socket arrangements (Miller and Sims, 1986). Pericytes regulate angiogenesis, playing a pivotal role in modulating gene expression of endothelial cells and in controlling astrocyte polarization (Armulik et al., 2010). Thus, by directly integrating endothelial and astrocyte functions, they regulate the integrity and permeability of the BBB also controlling the development of TJs (Daneman et al., 2010b). Collectively, endothelial cells, pericytes and associated smooth muscle cells contribute to the expression of a plethora of different cellular transporters, surface receptors, ion channels and efflux pumps that show a gradual phenotypic change along the arteriovenous axis of the adult mouse brain (Li et al., 2001; Calabria and Shusta, 2008; Armulik et al., 2010; Daneman et al., 2010a; He et al., 2016; Vanlandewijck et al., 2018). Altogether, pericytes, endothelial cells, astrocytes and neurons help form the BBB by directly communicating with each other and most importantly, pericyte loss is a central event in BBB damage and derangement that contributes to neurovascular dysfunction and neuronal injury, promoting also AD pathogenesis (Sengillo et al., 2013). Their degeneration boosts the development of AD as pericytes control multiple steps of AD pathogenic cascade, including elevation of Aβ deposition, tau pathology, and neuronal loss (Sagare et al., 2013). Taking all this into account, pericytes may thus represent and early and interesting therapeutic target, at the BBB level, to fight AD progression.

### Astrocytes

Astrocytes are the most abundant cell type in the brain and provide metabolic support to neurons (Rhea and Banks, 2019). They are closely interconnected to neurons and enter in touch with the endothelial cells of the brain capillaries through their end-feet, encircling the abluminal side of the blood vessels (Figure 2A), thereby regulating also fluids flow (e.g., blood and water content) across the CNS, including the function of ion and water channels as well as the clearance of toxins (Abbott et al., 2006; Obermeier et al., 2013). Along with the microglial foot processes and the basement membrane of the glia, astrocyte end-feet form the perivascular space border, which represents the last barrier to the entry of leukocytes and other infiltrating cells into the brain tissue (Man et al., 2007). These highly specialized cells also protect the BBB from insults and damages, and control immune reactions by recruiting parenchymal microglia *via* the release of specific soluble factors (Man et al., 2007). Parenchymal microglia triggers in turn the release of pro-inflammatory cytokines and chemokines, thus stimulating inflammatory and immune reactions as a defense mechanism (Town et al., 2005). Hence, astroglial cells are one of the primary targets for successful neuroprotective therapy as their alteration dramatically impact on neuronal survival and functionality. Another important role played by astrocytes at the endothelial cell level of the BBB is the regulation of surface transporter expression, the increase in TJ formation and the enhancement in the activities of many metabolic enzymes (Hayashi et al., 1997). Astrocytes, together with pericytes and extracellular matrix (ECM) help providing structural and functional support to the BBB (Obermeier et al., 2013), ensuring at the same time the interplay between the different NVU components. Overall, in virtue of all these key roles, including the interconnection between neurons and endothelial cells of the brain vessels and especially their intimate interaction with these latter cells, so as to influence their gene expression as well, astrocytes do represent a central target for NP surface functionalization strategies aimed at targeting the brain of AD patients.

### Neurons

Neurons are in close proximity to the endothelial cells of brain capillaries through the astrocyte end-feet, so that they can sense local alterations of the microenvironment and rapidly respond to regulate blood flow and microvascular permeability (Figure 2A; Zlokovic, 2008). As described above for the astrocytes, neurons can stimulate the microglia-mediated inflammatory response following vascular damages (Man et al., 2007). What’s more, in synergy with astrocytes, neurons take part in the regulation of TJ formation on endothelial cells and together with ECM modulate the expression of occludin (Savettieri et al., 2000). Therefore, during brain disorders such as AD, the BBB damage due to disruption of TJ integrity compromises the chemical composition of the neuronal milieu, leading to dysfunction of neuronal circuits with progressive synaptic loss and neuronal death (Zlokovic, 2008). To summarize, neurons cooperate with astrocytes at several levels to help controlling diverse mechanisms like maintenance of ion balance, immune and oxidative stress regulation, blood flow and water homeostasis as reviewed in detail in Liu et al. (2017).

In conclusion, loss of the BBB integrity, caused by the damage of any of its specific cellular component, leads to its functional failure and in turn to cerebrovascular dysfunction, neuroinflammation and neurodegeneration, contributing also to AD development (Desai et al., 2007; Obermeier et al., 2013; Sweeney et al., 2018). Therefore, even a disrupted and more permeable BBB can represent a serious challenge to brain-targeted drug delivery. However, the BBB alteration is at the same time a possible triggering factor and a consequence of AD (Erickson and Banks, 2013). It follows that, usually, the permeability and distribution of therapeutics across the AD BBB can be reduced compared to age-matched BBB in healthy subjects and this makes the BBB itself a possible therapeutic target of the disease (Banks, 2012).

### Different mechanisms of transport across the blood-brain barrier

**FIGURE 3:**
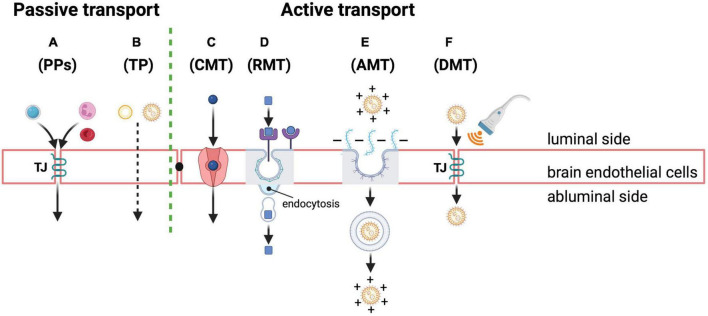
Passive and active transport mechanisms across the BBB as possible routes of NP entry and drug delivery into the brain. Passive transport through brain endothelial cells: **(A)** paracellular pathways (PPs) for passive diffusion of small water-soluble and polar substances (e.g., a small hydrophilic molecule in light blue) or cells (e.g., neutrophil and red blood cells depicted in pink and red in the cartoon, respectively) through the TJ structure (TJ in the cartoon); transcellular pathway (TP) **(B)** for passive diffusion of small non-polar and lipophilic molecules (e.g., micelles or lipid NPs in the cartoon). Active transport through brain endothelial cells: **(C)** carrier-mediated transcytosis (CMT) of small molecules through specific transporters (depicted in pink in the cartoon); **(D)** receptor-mediated transcytosis (RMT) of diverse molecules (e.g., neuropeptides, hormones and drug-loaded NPs depicted with square and round shapes in the cartoon) thanks to specific receptors (depicted in violet in the cartoon) that are highly expressed on the luminal side of endothelial cells; this pathway also represents the most exploited strategy to deliver drugs into the brain using polymeric NPs whose cell surface receptor can be captured by clathrin-coated vesicles (depicted in gray outline in the cartoon) that, following their internalization, release the final cargo into the abluminal side of the endothelium; adsorptive mediated transcytosis (AMT) of molecules **(E)** *via* electrostatic interactions between positive charged macromolecules, such as neuropeptides, drugs or drug-loaded NPs functionalized with cationic proteins/peptides (depicted in orange in the cartoon), and the negatively charged endothelial cell surface owing to anionic proteoglycans (depicted in light blue in the cartoon); after the invagination of the plasma membrane containing the extracellular molecules, the endocytic mechanism mainly relies on caveolae (depicted in gray and violet in the cartoon) for their transport and release; finally, a disruptive mediated transport (DMT) **(F)** also of NPs (orange) may occur through transient opening of TJ complexes between endothelial cells induced by different artificial methods and approaches, such as brain-targeted transcranial focused ultrasound (transducer depicted in gray and waves in orange in the cartoon) for local drug delivery. Created with BioRender.com.

As far as large molecular weight molecules (i.e., proteins and peptides) are concerned, the main routes for crossing the BBB are based on endocytosis through diverse specific and non-specific mechanisms of penetration in spite of the TJ sealing. These TJs and the absence of fenestrations are particularly crucial for preventing the paracellular diffusion of hydrophilic solutes, making brain endothelial cells significantly different from other endothelial cells present outside the brain. In more detail (Figure 3), there can be low levels of passive diffusion pathways across the TJs or through the transcellular lipophilic route and active pathways, mainly through transcytosis, such as carrier-mediated transcytosis (CMT), receptor-mediated transcytosis (RMT), and adsorptive mediated transcytosis (AMT), as also reviewed in He et al. (2018).

There are two types of *passive* pathways (Figures 3A,B), as follows:

(i) Some circulating cells, like infiltrating leukocytes, erythrocytes and neutrophils or water-soluble drugs and polar solutes can enter the BBB by harnessing or modifying TJ structure *via paracellular diffusion* (Figure 3A); this passive diffusion mechanism, which is though very limited for drug delivery purposes, is commonly used by highly lipid soluble substances with molecular weights less than 400–500 Da, or small hydrophilic molecules such as sucrose (Wong et al., 2019). Instead, substances and chemicals with neutral charge under physiological pH conditions show stronger permeability in the brain (Wong et al., 2019); (ii) the *transcellular lipophilic pathway* (Figure 3B) allows instead non-polar and lipid-soluble agents, such as small hydrophobic drugs, to cross the BBB (Wong et al., 2019).

On the contrary, expression of membrane receptors and transporters is necessary for *active transport* (Figures 3C–F) through the following transcytosis mechanisms:

Carrier-mediated transcytosis (CMT): This way of transport (Figure 3C) mainly involves small molecules such as glucose, amino acids and nucleosides that cannot diffuse through the cell membrane by themselves. These molecules can pass through the BBB by means of their specific transporters, such as GLUT1, LAT-1/CAT-1, and CNT2, respectively, in the direction of the concentration gradient (Pardridge, 2003; Chen and Liu, 2012). Interestingly, as to glucose transport into the brain, it has been shown that it relies on GLUT1 expression at the BBB cell surfaces, whose levels in mice overexpressing Aβ are downregulated. This downregulation turns out to be responsible for low sugar uptake and in turn for BBB breakdown, worsening AD cerebrovascular degeneration, neuropathology and cognitive functions (Winkler et al., 2015). Thus, GLUT1 certainly represents an interesting target for AD-centered nanodelivery of therapeutics.

Receptor-mediated transcytosis (RMT): It is the most common route exploited for delivering chemotherapeutic agents into the brain using the vesicular trafficking machinery of endothelial cells, as well as for the entry of NPs (Figure 3D) (Van Rooy et al., 2011; Chen and Liu, 2012). It enables large endogenous neuropeptides, enzymes, growth factors, transferrin and hormones, among the others, to penetrate the BBB harnessing their specific receptors that are highly expressed on the luminal side of endothelial as widely discussed in (Saraiva et al., 2016) and further explained, with representative examples, in section “Preclinical achievements of polymeric nanoparticles-mediated drug delivery in Alzheimer’s disease.” The same strategy can also be exploited to deliver drugs against AD into the brain, using surface-modified polymeric NPs as further described in section “Polymer functionalization strategies,” or even circulating exosomes (Wang et al., 2019). The most frequently used NP surface modifications include surfactants (e.g., the non-ionic surfactant and emulsifier polysorbate 80) or other ligands (e.g., MAbs, insulin, lactoferrin, and transferrin), which allow their binding to the corresponding receptors on endothelial cells that mediate the transcytosis process through membrane invagination. Upon binding, NPs are internalized through endocytic vesicles that carry out their final release in the abluminal side of the endothelium (Figure 3D; Saraiva et al., 2016). Most of the internalizations occurring through RMT are based on clathrin-coated vesicles of the brain endothelial cells (Mayor and Pagano, 2007). Notably, since the expression levels of the transferrin receptor (TfR) along with the TfR-dependent internalization mechanisms are still preserved and not affected in brain capillary endothelial cells of both mouse models of AD and human patients, TfR particularly represents an appealing target for NP-mediated drug delivery strategies (Bourassa et al., 2019).

Adsorptive mediated transcytosis (AMT): Beside receptor-mediated transcytosis, large proteins or neuropeptides can reach the brain from the bloodstream through the mechanism of AMT, with no involvement of protein receptors. Drug delivery of macromolecules can also be afforded *via* this transport route, which relies on the electrostatic interactions between the surface of positively charged NPs (functionalized for instance with positive polymers, cationic lipids, albumin) and negative charges naturally present on endothelial plasma membrane (due to the presence of anionic proteoglycans) of the BBB (Poon and Gariépy, 2007; Lu, 2012). Similar to RMT, the BBB entry occurs with an invagination of cell membrane holding the bonded molecule, followed by endosomal formation and transport from the luminal to the abluminal side, and finally with the exocytosis of the cargo (Figure 3E; Pulgar, 2019; Razzak et al., 2019). The principal endocytosis vesicles that take part to the AMT of extracellular molecules are caveolae (Mayor and Pagano, 2007). Remarkably, harnessing the capacity of positively charged albumin to transport drugs to the brain *via* AMT, the group of Dinarvand R. synthesized deferasirox and cationic albumin molecules containing novel nanosystems for brain drug delivery (Kamalinia et al., 2015). They used an AD rat model to assess the impact of such conjugated structures *in vivo*, with promising results. So, functionalization approaches of NPs based on cationic proteins and short amphipathic/cationic cell penetrating peptides (CPP), such as Syn-B-based vectors (i.e., a peptide derived from an antimicrobial peptide with high affinity for biological membranes) (Kokryakov et al., 1993) or TAT peptides (i.e., derived from the transcription activating factor of human immunodeficiency virus) (Vivès et al., 1997), are considered the most suitable strategies for brain drug delivery. Although AMT has higher capacity than RMT, it shows however, lower affinity for the ligand, thereby not representing a specific and ideal strategy for targeted drug delivery using NPs (Bickel et al., 2001). Yet, AMT has a clear advantage over RMT or CMT for drug delivery, that is the ability not to interfere with endogenous cellular functions as to RMT or CMT-mediated drug delivery do (Razzak et al., 2019).

Lastly, another mechanism that could be successfully used for NP-mediated drug delivery also in AD is based on the BBB disruptive mediated transport (DMD) (Figure 3F) through various means, which temporarily loosens endothelial cell junctions allowing drugs to pass through as more extensively reviewed by Saraiva et al. (2016). Some intriguing examples, also in mouse models of AD, come from the use of transcranial focused ultrasound that, combined with circulating NPs, could permit the transient opening of discrete sites on the BBB, showing large potential for AD treatment, such as a significant reduction in plaque burden (Jordão et al., 2013; Aryal et al., 2014). This kind of non-invasive method would facilitate penetration of therapeutics-loaded NPs into the BBB at desired points, otherwise inaccessible to drugs, by specifically targeting TJ complexes between endothelial cells of the BBB (Figure 3F).

In conclusion, targeting specific transport processes in the brain vasculature, tailoring and modulating the mechanisms by which NPs may cross the BBB and reach a specific brain area, may enhance drug transport through the BBB together with its therapeutic efficacy.

## Polymer functionalization strategies

**FIGURE 4:**
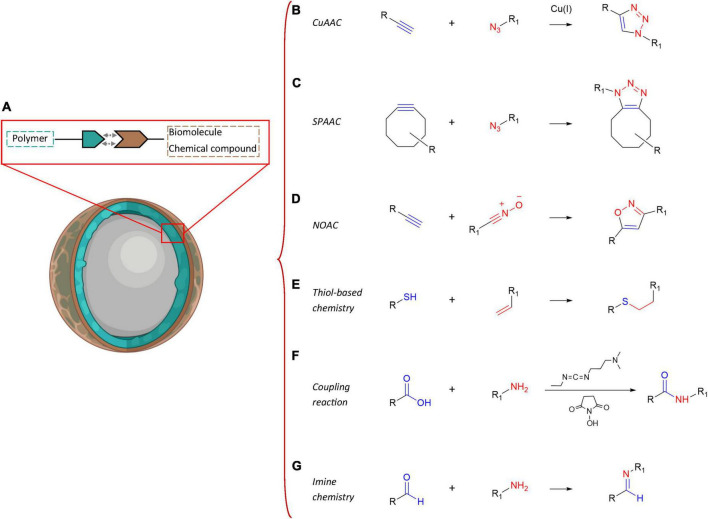
Summary of the possible chemical functionalization techniques of polymeric NPs. Scheme of the main chemical functionalization strategies of polymeric NPs **(A)** with biomolecules or specific chemical motifs: **(B)** copper-catalyzed azide-alkyne cycloaddition (CuAAC) reactions, **(C)** strain-promoted azide-alkyne cycloaddition (SPAAC) reactions, **(D)** nitrile oxide-alkyne cycloaddition (NOAC) reactions, **(E)** thiol-based chemistry, **(F)** coupling reactions, and **(G)** imine chemistry.

One of the most promising and cutting-edge strategies to deliver therapeutic drugs in a tailored manner into the brain of AD patients is by far the use of polymeric NPs (Figure 4A). These smart nanosystems provide a novel and safe tool for increasing the efficacy of pharmaceuticals administered parenterally *via* improved pharmacokinetics and biodistribution (Allen and Cullis, 2004). Polymeric NPs are block co-polymers made up of simple monomers of natural origin, such as albumin and polysaccharides, and synthetic origin, often present into the body and therefore largely biocompatible, biodegradable and easily excretable, as also reviewed in Calzoni et al. (2019) and Mukherjee et al. (2020). The most common polymers chosen for the synthesis of these NPs are, among others, chitosan, albumin, poly(butyl-cyanoacrylate), polylactic acid (PLA), PLGA, polyethylene glycol (PEG), and polyethylenimine (PEI). The inner structures of these macromolecules are characterized by chemical moieties suitable to form three-dimensional (3D) nanonetworks either *via* covalent crosslinking with other motifs of the same polymer backbone, resulting in one-polymer-based NPs or single-chain polymer NPs (Kröger and Paulusse, 2018), or reacting with other polymers and giving rise to heterogeneous NPs. Alternatively, the chemical groups that are inert toward the NP synthesis can be quantitatively converted into a broad range of other functional groups (Mauri et al., 2017). These groups can then address new combinations of polymers in a single nanoscaffold and promote the *ad-hoc* functionalization with targeting biomolecules to obtain selective and controlled release of therapeutics, proteins or nucleic acids to specific brain areas (Michaelis et al., 2006; Nagpal et al., 2010; Calzoni et al., 2019; Vismara et al., 2020; Mauri et al., 2021). Desired drug payloads can thus be entrapped, through diverse chemical reactions (i.e., covalent binding), by steric hindrance (i.e., non-covalent binding), bonded on the surface or physically dispersed/adsorbed within their meshes in a versatile manner (Kim et al., 2009; Ravichandran, 2009; Wais et al., 2016; Sotnikov et al., 2019; Graczyk et al., 2020; Mauri et al., 2021). Hence, it turns out that addressing the nature and chemical composition of the most suitable polymer is essential not only for effectively loading the nanocarrier with a cargo of therapeutic value, but also for selectively reaching the desired brain area or even the target cell type.

What’s more, several ligands can be conjugated to the surface of NPs in order to ease the BBB entry. These molecules are grouped into four different types: (i) ligands that adsorb proteins from the bloodstream able to interact directly with receptors or transporters expressed on the endothelial compartment of the BBB, such as T80 (Petri et al., 2007); (ii) ligands that directly bind BBB receptors or transporters, such as targeting ligands or Abs specific for the TfR, insulin receptor or glucose transporter (Ulbrich et al., 2009, 2011; Jiang et al., 2012; Gromnicova et al., 2013; Wiley et al., 2013; Shilo et al., 2014); (iii) ligands capable of increasing charge and hydrophobicity, such as amphiphilic peptides (Guerrero et al., 2010); and (iv) ligands able to enhance blood circulation time (e.g., PEG) (Choi et al., 2010).

Macromolecular drug-encapsulating NPs can be more efficiently delivered into the AD brain harnessing the AMT mechanism *via* their surface conjugation with cationic oligos, proteins or basic cell-penetrating peptides for an electrostatic interaction with the negative charged surfaces of the capillary endothelial cells (Lu, 2012; Zhu et al., 2019). The orthogonal chemistry represents the leading approach to perform selective conjugations between complementary functional groups, without undesired side-reactions. In NP design, the functionalization of polymer backbones with specific moieties enables the grafting of biomolecules and the generation of versatile carbon-based systems, thereby representing the closest approximation to biological tissues and materials suitable for controlled drug delivery. Several techniques for polymer functionalization and chemoselective grafting are discussed in the literature and implemented in an AD scenario. They can be classified in the following macro-categories: cycloaddition reactions (Figures 4B–D), thiol-based chemistry (Figure 4E), coupling reactions (Figure 4F), and imine chemistry (Figure 4G). Lastly, although polymer synthesis and functionalization are commonly used to enable safe drug transport and to facilitate its penetration through the BBB, some authors also exploited, at the same time, the polymer backbone itself as potential therapeutic agent in AD by leveraging its chemical characteristics. An interesting example was the use of sialic acid (SA)-modified selenium (Se) NPs conjugated with an alternative peptide-B6 peptide (B6-SA-SeNPs, a synthetic selenoprotein analog) in PC12 neuronal cells (Yin et al., 2015). In this work, the authors witnessed the high BBB permeability of these nanoformulations, thanks to the B6 peptide that acts *via* lipid raft-mediated and clathrin-mediated endocytosis (Liu et al., 2013a), and their ability to inhibit Aβ aggregation and disaggregate preformed Aβ fibrils (Yin et al., 2015). Furthermore, Se is long known to have important health effects in humans for redox regulation, being a potent antioxidant trace element showing protection against cognitive decline in animal models of neurodegenerative diseases (Schweizer et al., 2004). In the past, Se-NPs had already shown antioxidant properties through ROS scavenging activities both *in vivo* and *in vitro* (Zhang et al., 2001; Huang et al., 2003). Interestingly, Gupta et al. (2010) leveraged the therapeutic effect of the biphenyl ethers (BPEs) as known transthyretin (TTR) amyloid fibril inhibitors and disrupters to synthesize water soluble BPE-core shell CdSe/ZnS QDs. Using this QD system they showed the high binding affinity and disruption mechanism of the TTR fibers and the Aβ_42_ amyloids *in vitro* along with the detection ability of brain senile plaques in a transgenic AD mouse model. Also, polyphenol-based nanostructures, such as proanthocyanidins, tannins and tannic acid-functionalized NPs, could be potentially exploited as controllable, stimuli-responsive delivery systems in the brain due to their high drug release efficiency and stability (Bartzoka et al., 2017). The polymeric structures of tannins represent ideal scaffold building blocks for shell formation and nanocapsule assembly, and for safe delivery of hydrophobic active molecules, besides having potent antioxidant activity by themselves for potential therapeutic approaches in AD (Bartzoka et al., 2017; Türkan et al., 2019). As an example, Zhang et al. (2019) reported the use of metal-phenolic networks-coated gold NPs to inhibit β-amyloid fibril formation. The authors synthesized cobalt-tannic acid nanonetworks in which the cobalt coordination site facilitated its interaction with histidine and methionine residues in the amyloid-β peptides in synergy with tannic acid (Zhang et al., 2019).

However, there are only limited reports to date on the potential use of polymer chemical constituents or functional modifications as effective nanotherapeutics in AD, which we will consider a bit further in sections “Preclinical achievements of polymeric nanoparticles-mediated drug delivery in Alzheimer’s disease” and “Nanotheranostics” of the present review article.

### Cycloaddition reactions

This class of reactions represents the universal method for the synthesis of five-membered heterocycles as crosslinking point between the polymer chains and the biomolecules. Copper-catalyzed azide-alkyne cycloaddition (CuAAC), strain-promoted azide-alkyne cycloaddition (SPAAC) and nitrile oxide-alkyne cycloaddition (NOAC) are the most widely used approaches in biomedical scenarios (Figure 4). These reactions match the concept of click chemistry, exhibiting simple reaction conditions with high yields and high tolerance of functional groups. Indeed, these synthetic routes can be performed under mild conditions, insensitive to oxygen and water, proceed in benign solvents, show stereospecificity and generate inoffensive byproducts that can be removed without the use of chromatographic methods (Hein et al., 2008; Heaney, 2012; Avti et al., 2013; Yi et al., 2018; Meldal and Diness, 2020; Li K. et al., 2021).

In particular, CuAAC (Figure 4B) and SPAAC (Figure 4C) focus on the functionalization with azide and alkyne groups to link molecules such as proteins, peptides, carbohydrates, and fatty acids to other biomolecules or polymer matrices for generating biohybrid scaffolds with a plethora of improved properties. The azide-alkyne conjugation results in the formation of a triazole linkage, which is stable in physiological conditions. CuAAC involves the use of copper (Cu(I)) catalysts to enhance the reaction rate and regioselectivity toward the 1,4-substituted ring, avoiding the generation of a mixture of regioisomers that could affect the chemical and biological properties of the heterocycle (Du et al., 2021). However, the questioned toxicity of copper catalysts, even though well-established protocols of purifications (i.e., dialysis techniques) are available, has led to a strong demand of metal-free click reactions for biological applications. In this context, SPAAC represents the main alternative, where cyclooctyne derivatives are required to perform the bioconjugations, without additional catalyst. Nonetheless, the reaction is characterized by a slower rate compared to the CuAAC mechanism. Another biorthogonal cycloaddition can be conducted exploiting the interactions between reactive dipoles and alkyne motifs, as described in NOAC approach (Figure 4D; Heaney, 2012). The resulting linkage is an isoxazole, which is hydrolytically stable and shows similar isosteric character of amide and ester bonds. Additionally, the potential π-π stacking and hydrogen bonding with therapeutic payloads or biological molecules (i.e., peptides and proteins) can promote this type of crosslinking, which is another exploitable approach in the CNS for neuroactive candidates in the framework of neurological disorders (Pevarello et al., 1993; Belelli et al., 2019). Recently, cycloaddition was discussed by Duro-Castano et al. (2021): they functionalized polyglutamic acid (PGA) with azide and alkyne units, exceeding in the triple bond grafting by propargylamine reaction. The resulting nanostructure was obtained by polymer self-assembling and subsequent CuAAC-mediated crosslinking of the orthogonal star PGA chains. The residues of propargylamine exhibited neuroprotective effects resembling the effect of neuroprotective drugs such as Rasagiline (Amit et al., 2017). Auriemma et al. (2021) used CuAAC to decorate zwitterionic PCL-based nanoparticles with a radioligand (PBR28 derivative) selective for the 18 kDa translocator protein (TSPO), which is highly upregulated in the brain in pathological conditions. In this way, the authors assessed and monitored the microgliosis. In their work, Le Droumaguet et al. (2012) exploited the CuAAC chemistry to functionalize PEGylated poly(alkyl cyanoacrylate) nanocarriers with anti-Aβ_1–42_ antibody in order to perform a targeting approach toward Aβ_1–42_ peptides. The present strategy led to significant aggregation inhibition and toxicity rescue of Aβ_1–42_ in the field of therapies against AD (Le Droumaguet et al., 2012). Additionally, Büll et al. (2016) reviewed the click chemistry approach to functionalize polymer NPs with sialic acid mimetics capable to express a high affinity for Siglec-3, counteracting the amyloid-beta protein deposition and plaque formation in the brain and, as a result, the Alzheimer’s disease progression.

### Thiol-based chemistry

Thiol group is another example of functional moiety suitable for the conjugation among polymers and biomolecules (Figure 4E). The radical- and light-mediated reactivity of thiols with carbon–carbon double bonds ensures the NP functionalization, combining the advantages of click chemistry approaches with the benefits of a photoinitiated process activated at specific times and locations (Kade et al., 2010; Lowe, 2010). The thiol-based chemistry is characterized by quantitative yields, high reaction rates, mild reaction conditions (i.e., room temperature and aqueous solutions, including physiological buffers) and simple purification techniques of the final products and the formation of a single regioselective product. Representative reactions are free radical thiol–alkene and thiol–alkyne reactions, nucleophilic Michael addition and disulfide exchange. However, the most reported synthetic route in NP design for controlled drug delivery and selective targeting focuses on the crosslinking between thiol groups, present in proteins and peptides, and maleimide-modified polymers. For examples, the research group of Liu et al. (2013b) grafted thiolated lactoferrin, a natural iron binding protein, to PEG-PCL copolymer carrying maleimide units for developing NPs utilized to facilitate the nose-to-brain drug delivery of neuroprotective peptides; Martínez-Jothar et al. (2018) presented the conjugation of the peptide cRGDfK and the nanobody 11A4 (both containing a free thiol group) to maleimide functionalized PLGA NPs following the thiol-maleimide reaction, to tune the cellular internalization of NPs by cell surface receptor targeting. Finally, another report proposed the synthesis of PLGA nanocarriers decorated with PEG and glutathione (GSH), for the intracellular delivery of curcumin: the double functionalization resulted in an increased NP uptake by neuronal cells and, in particular, the GSH conjugation modified the NP internalization pathway, avoiding the lysosomal degradation and increasing the therapeutic fate for potential application in the treatment of AD (Paka and Ramassamy, 2017).

### Coupling reactions

The biochemical conjugation between polymeric NPs and biomolecules can be performed using coupling agents (Figure 4F). 1-ethyl-3-(3-dimethylaminopropyl)carbodiimide (EDC) and N-hydroxysuccinimide (NHS or N- hydroxysulfosuccinimide, sulfo-NHS) are commonly chosen to generate a zero-length crosslinker between the carboxyl groups and the amine motifs of the reagents. EDC couples NHS to carboxyl groups, forming an NHS-ester intermediate, which ensures an efficient conjugation to amines in physiological and buffer solutions. The formation of amide bond can be addressed using EDC only, however, the resulting O-acylisourea intermediate exhibits less stability in aqueous conditions: this reduces its reactivity toward amine moieties (due to hydrolysis) and the reaction yield. The carbodiimide chemistry is generally conducted in aqueous solution; however, the coupling mechanism could be carried out in organic medium using N,N′–Dicyclohexylcarbodiimide (DCC), which is the water-insoluble counterpart of EDC (Grabarek and Gergely, 1990; Conde et al., 2014; Yüce and Kurt, 2017; Wong et al., 2019).

This synthetic route is preferentially used to graft peptides or proteins to nanoscaffolds. Two representative examples come from the works of Mathew et al. (2012) and Bhatt et al. (2017), in which the authors functionalized PLGA NPs with the Tet-1 peptide to enhance the uptake by the neuronal cells and promote a targeted therapy approach in AD. An interesting strategy developed by the group of Picone et al. (2016) was based on the use of carbodiimide chemistry to conjugate a poly(N-vinylpyrrolidone) nanoscaffold to insulin: the obtained nanosystems were protected by protease degradation and capable to link to insulin receptors, trigger insulin signaling and promote neuroprotection effects against the dysfunction induced by amyloid-β. Interestingly, another research team recently developed simvastatin-chitosan-citicoline conjugated NPs for the co-delivery of the drugs and to counteract the potential secondary side-effects of simvastatin through the neuroprotective and psychostimulant action of citicoline (Mozafari et al., 2020).

### Imine chemistry

An alternative chemical route investigated in the bioconjugation of polymeric NPs is the oxime-based crosslinking (Figure 4G). The oxime bond is the result of the condensation reaction between a ketone or aldehyde and an aminooxy moiety to form a ketoxime or aldoxime, respectively. It exhibits high efficiency, chemoselectivity, formation in aqueous systems under mild acidic conditions and high stability toward hydrolysis. However, this chemistry is not widely used in NP synthesis for the treatment of AD. As a representative example of the chemistry, a research team from the Virginia Tech (Carrazzone et al., 2020) synthesized amphiphilic block copolymers of PEG and acrylate derivatives (2-(4-formylbenzoyloxy)ethyl acrylate, FBEA) and used O,O’-alkanediyl bis(hydroxylamine (BHA) as crosslinking agent in the core-forming block of the final micellar nanocarriers; additionally, they functionalized the hydrophobic portion of the polymer micelles with S-aroylthiooxime (SATO) H_2_S donors, which had been previously shown to induce neuroprotection and slow down progression of AD (Giuliani et al., 2013).

The most common method for preparing imine conjugation is the Schiff’s base reaction, which is classified as a group-specific reaction for aldehydes (Qin et al., 2013). Basically, the mechanism consists in the covalent linkage between an aldehyde (or a ketone) with a primary amine and the elimination of a water molecule. This synthetic route is generally chosen to develop gel-like scaffolds and, due to the mild reaction conditions, the drug encapsulation could be performed *in situ* during the formation of the nanomatrices, without affecting the therapeutic effect of the payload. Recently, this technique has been used to design polysuccinimide-based nanoscaffolds activated with ethylene diamine to address the decoration with oxidized EGCG (green tea polyphenol epigallocatechin-3-gallate): as a result, an improvement in the extracellular and intracellular anti-amyloidogenic effect of EGCG was detected, highlighting the potential of these nanocarriers compared to the administration of EGCG in its free molecular form in the prevention and curing of amyloid derived diseases (Debnath et al., 2016). In particular, the inhibition of amyloid fibrillation, the disintegration of amyloid fibrils and the decrease in amyloidogenic cytoxicity were estimated to be 10–100 more times effective after the administration of the engineered NPs. Lastly, Wang et al. (2020) revised the use of natural polyphenols for the design of nanocarriers, discussing the crosslinking between 3,4-dihydroxybenzaldehyde and 3,4,5-trihydroxybenzaldehyde with amine groups on the surface of cargo proteins to produce protein/polymer NPs for intracellular protein delivery. Furthermore, these nanoscaffolds could be decorated with boronate-modified polymers to improve the bioactivity of the proteins after their intracellular release.

## Effect of nanoparticles surface charge

Along with the grafting of specific biomolecules, another significant parameter affecting the BBB penetration is the NP surface charge. The presence of proteoglycans expressed on endothelial cells results in a negative charge of the BBB luminal side and, consequently, the development of positively charged NPs can improve the intracellular delivery of therapeutics exploiting the AMT mechanism (Hervé et al., 2008). The synthesis of this type of NPs can be performed in two manners: (i) using intrinsically positively charged polymers; (ii) conjugating protonated or positively charged motifs. The first method is based on the use of cationic polymers as building blocks, which, however, limit the physicochemical properties of the final nanoscaffolds to the inner features of the starting polymers. Indeed, generally, further functionalization is necessary to obtain an efficient targeting and intracellular drug release in AD. Positively charged polymers commonly used are chitosan (Rabiee et al., 2021), polyethyleneimine (Li Y. et al., 2021), cationic polymethacrylate-copolymers (Sahoo et al., 2019), and poly(amidoamine) (PAMAM), which has been recently harnessed to obtain cationic highly branched nanoscaffolds capable to cross the BBB (Srinageshwar et al., 2019). The second strategy (ii) has been successfully addressed using amine groups, chitosan or cationic albumin as decoration of NP surface.

Polystyrene (PS) NPs functionalized on their surface, for instance, with amino groups (PS-NH_2_), have long been used as safe nanocarriers to control proliferation and survival of leukemia malignant cells in anticancer drug strategies (Loos et al., 2014a). Besides, PS latex particles, presenting the advantage of having high versatility and ease of synthesis through a scalable process, can also be used as delivery vectors of RNA-based therapeutics (e.g., siRNAs, microRNAs and CRISPR-Cas9) in a wide range of applications, potentially including AD (Poon et al., 2016; Dowdy, 2017). Notably, owing to their biocompatibility and chemical inertness, PS-NPs exhibits no short-term cytotoxicity in a cellular environment, and being easy to synthesize and functionalize in various sizes, they are believed to be a good model platform for investigating bio-nano interactions (Loos et al., 2014b). Chitosan deposition on NP surface can be conducted either *via* physicochemical adsorption or covalent bonding between the polymer functional groups (i.e., -NH_2_ and -OH) and NPs (Annu et al., 2022). In a recent article, the potential of these two techniques has been described (Del Prado-Audelo et al., 2020). Briefly, the adsorption mechanism requires that NPs are added to a chitosan dispersion until reaching equilibrium. The polyelectrolyte behavior of chitosan promotes the generation of electrostatic interactions with the nanoscaffold, resulting in a physicochemical deposition of the polymer chains on the NP surface, through a process identified as electrosorption. In their research paper, Guo and Gemeinhart (2008) have shown the decoration of PLGA NPs with chitosan, demonstrating that the protonated amine groups are the main driving force of the adsorption and can lead to an external multilayer deposition around the NPs. The electrostatic attraction between positively charged chitosan and negatively charged surface of PLGA-based NPs was also exploited by the group of Qian et al. (2013); they synthesized nanoscaffolds capable of ensuring high stability to the encapsulated carmustine (BCNU), prolonging its *in vivo* circulation time so as to be used for targeted delivery to gliomas. In 2012, another research team (Mazzarino et al., 2012) designed poloxamer modified NPs composed of PCL, and decorated the final nanoscaffolds with chitosan. This functionalization introduced specific mucoadhesive properties; indeed, the resulting surface positive charge promoted the interaction with the negatively charged mucosal surface and enhanced drug absorption by opening the TJs between mucosal cells. Whereas, Wang et al. (2010) chemically crosslinked chitosan on PLGA nanocarriers through EDC activation of the NP carboxyl groups. In such a way, they obtained a functionalized nanosystem with enhanced cellular uptake that exhibited an efficient encapsulation of the coenzyme Q10, which turned out to be useful for the neuroprotection in a mouse model of AD. Additionally, several parameters could modify the physical and chemical grafting of the polyelectrolyte at the NP surfaces: pH, temperature, molecular weight, ionic strength and solvent quality are the main parameters to be tuned for performing an efficient chitosan decoration and generating positively charged NPs (Guo and Gemeinhart, 2008). Furthermore, the functionalization with cationized bovine serum albumin (BSA) represents an alternative approach to produce NPs exhibiting a cationic surface charge. In this case, the covalent bonding between BSA and the nanonetworks is the optimal route to achieve an efficient modification of the naïve NPs, compared to a physical crosslinking. The reaction is generally initiated by a coupling agent: as an example, Parikh et al. (2010) used EDC in acidic medium to activate and protonate BSA, prior to the addition to maleimide-functionalized PEG-PLA nanoscaffolds. The resulting cationic NPs were more efficiently internalized by the brain endothelial cells, compared to the uncharged counterpart, thereby confirming the higher affinity toward the negatively charged cells of the BBB and providing a targeted drug delivery system (Jallouli et al., 2007).

However, NPs exhibiting anionic surface charge are also used in AD treatment: in this case the starting polymers or the conjugated molecules are characterized by carboxyl, hydroxyl or carbonyl groups, and show an anionic nature in physiological environment. PEG functionalized poly(α,β-aspartic acid) (Anraku et al., 2017), PLGA (Amin et al., 2017), and hyaluronic acid (HA) (Jiang et al., 2018) are candidates for the synthesis of these NPs.

Overall, the administration of charged NPs need to be performed avoiding potential side effects related to the used NP concentration (Zhang et al., 2021a): nanocarriers with superficial cationic motifs exhibit a higher macrophage uptake and clearance by the mononuclear phagocyte system (MPS) compared to uncharged nanosystems, reducing the internalization in the targeted cells; whereas, anionic NPs could bind specific plasma proteins affecting the receptor binding and BBB transcytosis. Moreover, high concentration of ionic NPs could disrupt the BBB with consequent toxic effects on brain microvasculature endothelium (Lockman et al., 2004).

## Preclinical achievements of polymeric nanoparticles-mediated drug delivery in Alzheimer’s disease

Most of the current conventional therapeutics used in the treatment of neurodegenerative disorders, including AD, are based on high molecular weight (Mw ≥ 500 Da) Abs, peptides and proteins, which are unable to go through the BBB by themselves (Mukherjee et al., 2020). Thus, owing to the large surface area to volume ratio of NPs and thanks to the wide range of surface functionalization that can be done through different chemical groups, these nanocarriers can provide a great variety of interesting possibilities to overcome the BBB obstacle, with better results compared to traditional therapeutic approaches. Another important advantage is the NP capacity to slowly release a drug to therapeutic doses into the brain, thereby reducing peripheral toxicity (Zhang et al., 2018). To date, however, most of the research efforts on NP-mediated delivery of therapeutics against AD have been focused on animal models, with just a few drugs tested for their brain-targeted delivery by nanocarriers and limited only to lipid NPs, which are supposed to reach the essential prerequisite for clinical-stage mass production, as highlighted in He et al. (2018). Major limitations, before their large-scale clinical application, are still represented by: (i) appropriate quality control for CNS biocompatibility and safety, including their complete, efficient and fast clearance/reabsorption, (ii) establishment of the ideal mechanism of NP-mediated drug delivery into the brain, and (iii) a careful consideration of the multiple factors that may affect NP efficacy into the brain microenvironment, such as the ideal material, size and shape, surface modification.

Since the BBB is made up of a layer of endothelial cell junction complexes, strategies that aim to unhinge these TJs and AJs would allow NPs to enter the brain circumventing the BBB. Polymeric NPs can either breach the BBB *via* surface functionalization, for instance using Abs directed against endothelial cell surface receptors leveraging the RMT route, or negatively charged on their surface, for intravenous applications, as described above in sections “Different mechanisms of transport across the blood-brain barrier” and “Effect of nanoparticles surface charge” and also reviewed in Saraiva et al. (2016). In the former example, chitosan NPs were conjugated with specific Abs against the TfR-1, expressed on brain endothelial surface, to mediate their transcytosis and deliver neuroprotective peptides into the brain of injured mouse models (Yemisci et al., 2015). In preclinical mouse models of Parkinson’s disease, poly(butyl-cyanoacrylate)-based NPs loaded with dopamine have been successfully used as an efficient drug delivery system (Jahansooz et al., 2020) and could be also exploited for traversing the BBB of AD mouse models. Interestingly, previous findings demonstrated that these NPs loaded with BBB-impermeable drugs and imaging probes, can enter the brain *via* a receptor-mediated endocytosis mechanism. When they were coated with the T80, once entering the bloodstream, they absorbed apolipoproteins on their surface, thus mimicking lipoprotein particles, which helped them to be taken up by a low-density lipoprotein receptor through the BBB of various mouse models, including AD ones (Kreuter et al., 2002; Koffie et al., 2011). Brain imaging analysis further demonstrated that these NPs made of poly(n-butyl cyanoacrylate) dextran polymers coated with T80 complex with plasma apolipoprotein E to facilitate BBB crossing (Koffie et al., 2011). In the AD context, it has been shown that the uptake of the anti-Alzheimer’s drug rivastigmine was highly increased when bound to poly(n-butylcyanoacrylate) NPs coated with 1% T80 *vs.* the free drug intravenously injected in AD rats (Wilson et al., 2008a). Similar results were also achieved in the administration of rivastigmine and tacrine through chitosan-based NPs with 1% T80 coating in rats, for their potential use in the treatment of AD (Wilson et al., 2010, 2011). As a general consideration, nanoparticles made with hydrophilic polymers or coated with hydrophilic surfactants, such as the ones discussed above, circulate for a longer time in the blood, as they are able to partially escape the uptake of the reticuloendothelial system of the liver (Gaur et al., 2000). This highly desired feature, together with the high biocompatibility and stability of NPs in biological environments, can increase the drug availability and sustained release to the target site, including the brain.

Another interesting intervention strategy that is being recently explored in the search for alternative cures against AD is represented by nanoformulated natural products, such as phytochemicals, through the brilliant merging between medicinal plants and nanotechnology (Jadhav et al., 2017; Woon et al., 2021). One of the major needs related to the use of phytotherapeutics in AD is indeed the reduction of severe side effects of current drugs while maintaining their medicinal potentials and efficacy (Varshney and Siddique, 2021). In this context, also polymeric NPs have been employed to efficiently deliver plant-based therapeutics to the brain by crossing the BBB with some promising results, as exhaustively reviewed by Ovais et al. (2018). The most common action mechanisms of plant-derived agents against AD, ranges from anti-amyloidogenic activity as well as inhibition of tau hyperphosphorylation and tangle clearance, to the anti-inflammatory potential within the neuroglia and protection against free radical-induced neurodegeneration, thereby exhibiting important neuroprotective effects by enhancing the memory and learning process that normally regress in patients (Baum et al., 2008; Ahmad et al., 2016; Ali et al., 2017; Ayaz et al., 2017a,b). Various natural plant products and secondary metabolites including flavonoids, terpenoids, alkaloids, tannins and phenols are used to reduce the progression of AD and to mitigate the disease-related synthoms (Varshney and Siddique, 2021). Among several examples of preclinical applications, nanodelivery of curcumin against aggregation of the tau protein and β-amyloid plaques is considered one of the most attractive. For instance, Tsai et al. (2011) studied the high capacity of curcumin-loaded PLGA NPs to penetrate the animal cerebral cortex and hippocampus, while the group of Cheng et al. (2013) tested a stable curcumin NP formulation made of PEG-PLA co-block polymer and polyvinylpyrrolidone (PVP) in an *in vitro* BBB model and in the AD mouse model Tg2576 orally administered for 3 months; their nanocurcumin formulation was able to lower amyloid plaque burden in AD mouse brains compared with controls and in turn to improve cue and working memory than ordinary curcumin or placebo in different behavioral tests.

Yet another interesting preclinical study was based on the injection of curcumin-conjugated nanoliposomes, with curcumin exposed at their surface, into the hippocampus and the neocortex of the double transgenic APP/PS1 mouse model of AD; the authors exploited the behavior of curcumin that, being a fluorescent molecule and having high affinity for the Aβ peptide, was able to specifically label Aβ deposits *in vivo* both in AD mouse brains and human post-mortem AD brains as well as to downregulate the secretion of the amyloid peptide *in vitro*. Thereof the authors concluded that such curcumin nanoformulations could be used for both diagnosis and targeted drug delivery in AD patients (Lazar et al., 2013).

Besides curcumin, the potent anti-inflammatory polyphenolic phytochemical, resveratrol, found in many plants, is considered a potential nutraceutical agents useful against AD. Resveratrol showed antioxidant activity and anti-Aβ formation features both *in vivo*, in Tg2576 mice, and *in vitro* through different molecular mechanisms, including inhibition of β-amyloid-induced neuronal apoptosis *via* regulation of the silent information regulator 1 (SIRT1)-Rho-associated kinase 1 (ROCK1) signaling pathway in PC12 cells and by promoting non-amyloidogenic processing of APP, and thus mitigating Aβ neuropathology in AD (Wang et al., 2006; Ladiwala et al., 2010; Feng et al., 2013). However, recent AD clinical trials with natural compounds, including curcumin and resveratrol as well, failed in different phases to successfully treat AD (Kanubaddi et al., 2018). This has been partially due to the low aqueous solubility, poor bioavailability, and ineffectiveness against the disease mainly because of the low penetration of resveratrol and other natural molecules across the BBB; whereby the need of alternative NP-based formulations for improving pharmacokinetic properties, bioavailability, neural targetability and the great clinical potential of these nutraceuticals (Summerlin et al., 2015; Kanubaddi et al., 2018).

Over a decade ago, it was already exploited the α-secretase inducing ability of EGCG, the predominant green tea polyphenol, against brain β-amyloid plaque formation in mouse models of AD using nanolipidic EGCG particles based on amphiphilic block copolymers (Smith et al., 2010). The same authors had previously showed a novel mechanism of action of the EGCG, which was able to promote the non-amyloidogenic processing of the APP in AD mice (Rezai-Zadeh et al., 2005; Obregon et al., 2006). Thus, in their more recent preclinical work, Smith et al. (2010) improved the oral bioavailability of the nanoformulated EGCG *in vivo* and found an increased α-secretase enhancing ability of this encapsulated polyphenol *in vitro*, using Swedish mutant APP overexpressed N2a (SweAPP N2a) cells, compared with free EGCG. In fact, they stated that, unlike the excellent therapeutic results observed in mouse models of AD, translating them to a human clinical trial would be tough because of the limited oral bioavailability and without an efficient systemic delivery tool, which could be now potentially represented by their nanolipidic particle complexes.

As already described in section “Alzheimer’s disease,” one of the main etiologic factors involved in the development and progression of AD is the oxidative stress due to the production of ROS generated by various molecular mechanisms and cellular metabolic reactions (Smith et al., 1995; Perry et al., 1998; Markesbery and Carney, 1999). There is a great deal of evidence that a major pathogenic role in oxidative stress is played by transition metals, such as iron, copper and zinc, which catalyzes oxidation reactions also in the brain and their imbalance was frequently detected in brain tissue of AD patients thereby representing an interesting therapeutic target in AD treatment (Gutteridge, 1994; Casadesus et al., 2004). For instance, copper, zinc, and iron, whose excessive concentration is toxic in the body, have been found at significantly elevated concentrations in senile β-amyloid plaques and neurofibrillary tangles of AD patient brains compared with healthy controls (Lovell et al., 1998; Sayre et al., 2000). Hence, the strategy of treating AD with copper and zinc chelators such as clioquinol was shown to be able to reverse Aβ deposition and in turn to improve cognitive decline and behavior in AD transgenic mice and patients (Cherny et al., 2001; Ritchie et al., 2003). Nevertheless, clioquinol is potentially toxic and, alike other chelating agents, does not cross the BBB easily and in therapeutic amounts (Andersen, 2004; Liu et al., 2009). Indeed, similar to clioquinol, therapeutic chelation of transition ions using desferrioxamine (DFO) or ethylenediaminetetraacetic acid (EDTA) was able to improve symptoms and slow down the progression of dementia in AD patients (Casdorph, 1981; McLachlan et al., 1991) although the drawback of possible noxious side effects due to chelator toxicity along with the issue of their low BBB passage remain to be solved. Therefore, the development of improved and safer NP-mediated delivery methods is strongly desirable to keep going with the AD chelation therapy in a more effective and safe manner. What’s more, this strategy may allow the chelator to leave the brain once the target metal has been bound to the NP-conjugated chelating agent (Liu et al., 2009). So far, only very few attempts have been made in this direction, such as the use of polystyrene NPs with carboxyl groups on their surface to conjugate the hexadentate chelators 3′-aminopropyl-3-hydroxyl-4-pyridinone (MAPHP) or DFO for excess iron removal. The great advantage of this method is that also toxic bidentate or tridentate iron chelators can be modified by conjugation with NPs into less toxic hexadentate chelators that hold high kinetic stability, without affecting their iron binding capacity (Hider, 1994; Shi et al., 1997). Then, by changing the surface properties of the MAPHP-particle system through coating surfactants (e.g., T80), by overcoating with ApoE, B, A-I or by complexing with iron, the system could mimic the behavior of the apolipoprotein E- (ApoE) and apolipoprotein A-I (ApoA-I)-NPs, respectively, so as to cross the BBB and take the excess metals out of the brain with very limited toxicity (Davson and Segal, 1995; Alyautdin et al., 1998; Kreuter et al., 2002). However, further *in vitro* assays and preclinical studies with this NP-delivered metal chelator approach are warranted to improve their targeting capacity to the brain as well as to estimate the level of BBB crossing, chelation efficiency and the related toxicity before clinical applications.

As far as the surface charge is concerned, thoughtful considerations must be taken before NP administration for therapeutic purposes. It has indeed been shown that neutral and low-anionic (between −1 and −15 mV) NPs have no or poor effect on BBB integrity, unless given at high concentrations. Whereas, highly positively charged NPs (i.e., with high zeta potential over 15 mV) trigger immediate toxic effects by damaging the BBB also at low dosage (Lockman et al., 2004). However, another work showed that neutral and cationic porous maltodextrin-based NPs (with moderate to high-positive zeta potentials) under physiological conditions showed similar and higher uptake into endothelial cells, by transcytosis, than anionic NPs, turning out to be efficient drug delivery systems (Jallouli et al., 2007). Finally, another important consideration regards NP pharmacokinetics after their *in vivo* administration. Longer circulation times are preferable for brain delivery strategies, with neutral and zwitterionic NPs showing slower clearance from the bloodstream compared with negatively and positively charged NPs, which instead presented shorter half-lives (Arvizo et al., 2011).

Altogether, polymer-based NPs represent a promising and competitive alternative to other conventional carbon-based and bio-based nanostructures eligible for potential clinical applications, such as fullerene, carbon dots (CDs) and nanotubes as well as liposomes (Bilal et al., 2020; Faiyaz et al., 2021).

Fullerene (particularly in its endohedral form) can be used for diagnostic aims in magnetic resonance imaging (MRI), or as radioactive tracer for CNS imaging (Du et al., 2018); additionally, its chemical structure exhibits a significant inhibitory effect on the formation of β-sheets of the Aβ and interferes in the oxidative stress in AD: these outcomes could be ascribed to the hydrophobic interactions and aromatic-stacking interactions present between the hexagonal rings and the peptides/biomolecules. Regarding fullerene, targeted cell interactions are addressed through specific peptide sequences linked to the carbon structure.

Multi-walled carbon nanotubes (MWCNTs), a sub-family of fullerenes, can also serve to intracellularly deliver peptides, nucleic acids or other therapeutics. A preclinical evidence of the MWCNTs potential was discussed by Lohan et al. (2017), where the nanostructures were decorated with polysorbate together with phospholipids and berberine (an isoquinoline alkaloid), encapsulated in the resulting nanocarrier: the formulated biomaterial showed to play a neuroprotective role in AD progression.

Carbon dots, thanks to their abilities to penetrate the BBB and the excellent photoluminescence may represent promising nanocarriers for drug delivery in neural compartments and for potential use as AD therapy, by inhibiting oxidative stress, neurotoxicity as well as for restoring nerve damage and maintaining neuronal morphology according to the released payload (Kuang et al., 2020; Zhang et al., 2021b). Also in this case, the preferential functionalization of CDs involves the use of biomolecules, such as amino acids and aptamers, to achieve promising results toward a preclinical AD application: for example, a Korean research team (Chung et al., 2020) has decorated carbon dots with aptamers to selectively targeting Aβ_42_ species whereby highlighting their therapeutic functions. These CDs can thus be potentially used as light-powered nanomodulators to spatiotemporally suppress toxic Aβ aggregation both *in vitro* and *in vivo.*

Lastly, liposomes, due to their inner composition, represent a pivotal approach against AD (Ross et al., 2018). However, coating with polyoxyethylene, PEG, cholesterol, polyvinylpyrrolidone, polyacrylamide, distearoyl phosphatidylcholine is generally required to improve the drug release performances thanks to a sustained circulation time and protection from the immune system (Cui et al., 2011; Faiyaz et al., 2021). A list of surface chemical functionalizations of polymeric NPs with their potential therapeutic advantages in the AD scenario is reported in Table 1.

**TABLE 1:** Summary of functionalized polymeric NPs and their potential advantages.

Functionalization	Polymer	Reaction	Advantages	References
Abs anti Tfr-1	Chitosan	Electrostatic interaction	To mediate transcytosis and deliver neuroprotective peptides into the brain of injured mouse models	Yemisci et al., 2015
Abs anti-Aβ_1–42_	PACA	CuAAC	To enable specific targeting to Aβ_1–42_ peptides for inhibiting their aggregation and for rescuing their toxicity effect	Le Droumaguet et al., 2012
Amino groups	PS	Free-radical polymerization in emulsion	To modulate mTOR signaling	Loos et al., 2014a
Azide and alkyne units	PGA	CuAAC	To increase drug transport across the BBB showing neuroprotective effects similar to those of neuroprotective drugs	Duro-Castano et al., 2021
B6 peptide	PEG-PLA	Thiol- maleimide	To increase BBB permeability *via* lipid raft-mediated and clathrin-mediated endocytosis To inhibit Aβ aggregation and disaggregate preformed Aβ fibrils	Liu et al., 2013a; Yin et al., 2015
Boronate	Polyphenols	Catechol-boronate complexation	To improve the bioactivity of delivered proteins after their intracellular release	Wang et al., 2020
BSA and cationized BSA	PEG-PLA	EDC	To increase their internalization by the brain endothelial cells. cBSA accumulation is much higher than BSA-NPs	Parikh et al., 2010
Carboxyl groups	PS	Free-radical polymerization in emulsion	To increase internalization time in macrophages	Loos et al., 2014b
Chitosan	PLGA PCL- triblock surfactant poloxamer (PEO–PPO–PEO)	Electrostatic interaction Physical interactions	To increase plasma stability, drug efficacy and safety To increase plasma stability, drug efficacy and safety	Guo and Gemeinhart, 2008; Mazzarino et al., 2012; Qian et al., 2013
Curcumin–phospholipid conjugate	DPPC/Cholesterol + DPS-curcumin	Michael addition using DPSH and DIPEA	To specifically label Aβ deposits *in vivo* both in AD mouse brains and human post-mortem AD brains	Lazar et al., 2013
EGCG (green tea polyphenol epigallocatechin-3-gallate)	Poly succinimide	Schiff’s base	To promote the non-amyloidogenic processing of the APP in AD mice	Rezai-Zadeh et al., 2005; Obregon et al., 2006
Oxidized EGCG	Poly succinimide	Schiff’s base	To increase extracellular and intracellular anti-amyloidogenic effect by 10–100 times	Debnath et al., 2016
Hydroxypropyl β-cyclodextrin	PEG-PLA co-block and PVP (as stabilizer)	Physical interactions	To cryoprotect the drug (curcumin) during the NP freeze drying, preserving the therapeutic activities of the nanoformulates; suitable to reduce Aβ plaque burden in AD mouse brains and to improve cue and working memory	Cheng et al., 2013
Insulin	Poly (N-vinyl pyrrolidone)	EDC	To protect from protease degradation and bind to insulin receptors	Picone et al., 2016
Lactoferrin	PEG-PCL	Thiol-maleimide	To facilitate the nose-to-brain drug delivery of neuroprotective peptides	Liu et al., 2013b
PEG and GSH	PLGA	Thiol-maleimide	To increase the uptake by neuronal cells; the presence of GSH has a better neuroprotective effect; to avoid lysosomal degradation and increase therapeutic fate of delivered drugs	Paka and Ramassamy, 2017
Peptide cRGDfK and nanobody 11A4	PLGA	Thiol-maleimide	To tune cellular internalization by targeting cell surface receptors	Martínez-Jothar et al., 2018
Poly (α, β-aspartic acid) with glucose conjugated	PEG	Self-assembling	To increase and selectively control the transport of bioactive substances into the brain *via* GLUT1	Anraku et al., 2017
Polysorbate 80	Poly(butyl cyano-acrylate) Chitosan	Physical interaction Physical interaction	To facilitate BBB crossing and increase drug uptake To facilitate BBB crossing and increase drug uptake	Wilson et al., 2008a,Wilson et al., 2010, 2011; Kreuter et al., 2002; Koffie et al., 2011
Proteins	Polyphenols	Schiff’s base	To enhance intracellular protein delivery	Wang et al., 2020
Radioligand (PBR28 derivative)	PCL	CuAAC	High capacity of brain penetration and high selectivity for 18 kDa translocator protein (TSPO) for monitoring microgliosis	Auriemma et al., 2021
SATO	FBEA	Oxime chemistry	Neuroprotection and slow-down of AD progression	Carrazzone et al., 2020
Sialic acid mimetics	PLGA	EDC	To improve binding ability to Siglec-3, counteracting the Aβ protein deposition and plaque formation	Yin et al., 2015; Büll et al., 2016
Simvastatin and citicoline	Chitosan	EDC	To counteract the potential secondary side-effects of simvastatin through the neuroprotective and psychostimulant action of citicoline	Mozafari et al., 2020
Tet-1 peptide	PLGA	EDC	To enhance the uptake by neuronal cells and promote a neuronal- targeted therapeutic approach	Mathew et al., 2012; Bhatt et al., 2017
Trimethylated chitosan (TMC)	PLGA	EDC	To enhance drug delivery and cellular uptake with low toxicity	Wang et al., 2010

## Nanotheranostics

Development of theranostic platforms to investigate and modulate neuroinflammatory and neurodegenerative processes occurring during neurodegenerative disease progression, such as in AD, represents quite a novel and appealing strategy holding tremendous potential. The use of a traceable cell-targeted nanovector platform loaded with a therapeutic drug, allows scientists to gain better insights into the complexity of neuronal and glial cell responses paving the way, at the same time, for more efficient therapeutic approaches.

Taking into account the amyloid β-protein oligomer paradigm in the etiology of AD, as early and pathognomonic predictors of the disease, some interesting works reported the existence of several challenges that must be faced during Aβ oligomer detection *in vitro* as well as *ex vivo* through different techniques of analysis, including SDS-PAGE rather than surface enhanced laser desorption/ionization time-of-flight mass spectrometry (MS) or size exclusion chromatography, which may give rise to misleading data (Marina et al., 2003; Bitan et al., 2005; Hepler et al., 2006; Gallagher, 2012; Watt et al., 2013). This can be due to Aβ oligomers undergoing structural changes because of the extraction method used, thereby artifactual dissociation and formation of oligomers can easily occur before and after analysis in biological fluids and tissue extracts, thus requiring thoroughly interpretation of results (Hayden and Teplow, 2013). From this and other considerations rises the necessity to track *in vivo* the neurodegenerative process from its early onset through alternative and trustful diagnostic tools than traditional ones. In such a context, nanotheranostics may represent a smart strategy for precocious AD diagnosis and, hopefully, in furtherance of potential therapeutic treatments. In this context, the rational assembling of polymeric NPs is still challenging. Indeed, the literature proposes several strategies to use polymer chains as surface functionalization of inorganic nanomaterials, such as metal NPs, radionuclides and QDs. The polymer coating is generally addressed by choosing among a wide range of natural and synthetic macromolecules, such as dextran, chitosan, cyclodextrin, PEG, polyvinyl alcohol (PVA), polydopamine (PDA), polysaccharides, PEI, PVP, and PAMAM (Azria et al., 2017; Wu et al., 2020; Gopalan et al., 2021). The polymer conjugation occurs *via* electrostatic interactions, adsorption mechanism or orthogonal grafting between complementary chemical reactive groups. Gold (Zhao et al., 2022), silver (Ahmad et al., 2017), superparamagnetic iron oxide (SPIONs) (Amiri et al., 2013), gadolinium-based NPs, and QDs (Gupta et al., 2019) represent the most common inorganic core decorated with polymeric backbones (Bilal et al., 2020). Gold NPs exhibit good biocompatibility, low toxicity, interesting optical detection and imaging properties; these inorganic nanosystems can be synthesized following simple protocols and are able to cross the BBB by connecting various ligands or altering their sizes. Moreover, as discussed in an interesting work of 2017 (Muller et al., 2017), gold NPs take action in the prevention of oxidative stress, cognitive deficits and show anti-inflammatory effect in rat models of AD. In another report, the PEGylation of these nanocarriers loaded with anthocyanins resulted in an enhanced efficacy in the prevention of neurodegenerative disorders such as AD, compared to the uncoated specimen both *in vivo* and *in vitro* (Kim et al., 2017). Alternatively, chitosan has been extensively used as NP decoration for sensing applications, obtaining biosensors capable of measuring the level of plasma acetyl choline in patients suffering from AD (Yadav et al., 2018). Silver nanocarriers are usually tagged with specific peptides, such as Aβ oligomer binding peptides, to detect Aβ oligomers in the AD brain; however, the NPs can be anchored to β-cyclodextrin to amplify the electrochemical signal detection or even stabilized with PVP (Kim et al., 2018; Fehaid and Taniguchi, 2019; Gopalan et al., 2020). Mirsadeghi et al. (2016) decorated SPIONs with PEG characterized by different molecular weight and terminal reactive groups (i.e., -NH_2_, -COOH, and -OH), obtaining charged coatings. As a result, NPs were able to influence the Aβ fibrillation accelerating or inhibiting the process; instead, de la Torre and Ceña (2018) reviewed the decoration of magnetic NPs with polysaccharides or PEG to protect the magnetic core and preserve the diagnostic and imaging functionalities for diagnostic and therapeutic aims in different models of AD.

Regarding QDs, their use in nanomedicine is favored by their unique optical properties that provide high sensitivity, stability and selectivity at a nanoscale range. For these reasons, QDs are promising candidates for *in vivo* detection of deposits or aggregates of amyloid in AD brain (Cantore, 2019). Cationic poly(fluorene-*alt*-phenylene), PEG and chitosan (Ahmed and Ikram, 2017; Yuan et al., 2020) are mainly used as polymeric crosslinked moiety of QDs *via* hydrothermal treatment, coupling reaction or ultrasonic treatment, to design non-toxic and multifunctional probes. These conjugated polymer-QD hybrid materials can find application as innovative theranostic platforms in different biomedical contexts.

For all the above advantageous characteristics and potentialities, the design of polymer-based NPs represents an innovative strategy to synthesize also theranostic agents for imaging and probing in neurodegenerative diseases. For instance, co-assembled NPs of chitosan and HA crosslinked with glutaraldehyde have been developed as theranostic nanosystems for targeting Alzheimer’s β-amyloid (Wang et al., 2021). The imine bond between the two polymer chains gives rise to nanoscaffolds characterized by a selective recognition toward negative Aβ oligomers, resulting in a red fluorescence emission upon interaction with the abnormal proteins. Furthermore, NPs inhibited the formation and aggregation of Aβ fibrils, counteracting the AD degeneration. These features prove the potential of the polymer NPs as nanoagents for the diagnosis and treatment of AD. Alternatively, dye molecules emitting far infrared light or with fluorescent properties are chosen as imaging markers in the development of useful theranostic nanocomposites both *in vitro* and in preclinical animal models of ROS-related diseases, including AD (Peng et al., 2015; Li et al., 2018; Yan et al., 2019).

## Conclusion and future perspectives

Dementia including AD are the fifth leading cause of death globally, and the overall burden of these diseases is expected to rise in the next decades because of the increase in the general population ageing (Nichols et al., 2019). Being a complex and multifactorial disease, which is caused by both genetic and environmental factors, AD is very difficult to treat. Moreover, most of the clinical trials fail to enter the market with novel effective drugs, with just 1 out of 100 new molecules developed against AD that reaches the pharmaceutical market (Nichols et al., 2019; Cummings et al., 2020). Hence, the lack of promising and curative treatments will heavily affect the healthcare systems worldwide (Cummings et al., 2020). To date, most of the clinical and experimental studies have experienced a very high failure rate in the development of new effective therapies against AD. This has been due to our poor understanding of the mechanisms underlying AD, wrong choice of potential targets and development of ineffective drugs as well as to the difficult administration route through the BBB (Mangialasche et al., 2010; Revi, 2020). One of the crucial reasons is also having addressed molecular targets and key neuropathological changes acting too late in the disease, such as Aβ plaques and hyperphosphorylated tau protein deposits (Godyń et al., 2016). Thus, it is pivotal that novel clinical and preclinical studies on animal models of AD will intervene as preventive treatments with more effective therapies and will focus on early targets that play a clear role in the development of the disease (e.g., cholinergic drugs which can inhibit the enzyme that degrades acetylcholine, and intracellular clearance mechanisms, such as autophagy) (Dubois et al., 2014; La Barbera et al., 2021) harnessing, at the same time, the efficacy of polymeric NPs as promising drug delivery systems.

In the last decades, therapeutic nanomedicine has started to build momentum also in the field of neuroscience, with a potential impact on human health and for enabling multidisciplinary research (Barz, 2015; Derakhshankhah et al., 2020). For instance, the application of nanotheranostics may potentially improve, at the same time, diagnosis, prevention and treatment of various neurological disorders including AD, as reviewed in (Mukherjee et al., 2020). Smart functionalization of polymer-based nanoscaffolds with specific chemical or biological motifs guarantees *ad-hoc* design of nanocarriers for targeted approaches. Indeed, the desired grafting could be addressed through chemical or physical routes, which give rise to orthogonal modifications of the reactive groups of polymer backbone or side chain, thus minimizing the formation of side-products and meeting the criteria of cytocompatibility and biodegradability that are highly requested in AD treatments. The rational choice of the starting materials and their functionalization offers the opportunity to obtain a plethora of nanocarrier configurations, overcoming the main limitations associated to the use of conventional therapeutic methodologies.

In summary, the main benefits provided by nanoformulated drug carriers are the possibility to increase the therapeutic index of compounds with higher and more targeted efficacy in a controlled fashion, through inner and surface chemical modifications; nanocarriers have also shown protection of the drug payload from degradation into a biological environment, eventually minimizing potential side effects for some of the symptomatic treatments approved by the FDA (Ravichandran, 2009; Talekar et al., 2015; Wais et al., 2016; Bukhari, 2021; Cano et al., 2021). Furthermore, nanotherapeutics can enhance solubility of hydrophobic drugs and absorption of poorly soluble molecules thereby resulting in their augmented bioavailability and increased brain uptake (Ravichandran, 2009; Cano et al., 2021). Due to their advanced features, these nanoformulations may help overcome current obstacles for delivering therapeutically active pharmaceuticals into the brain for the purpose of treating neurodegenerative diseases. Although such nanoparticle-based systems may offer a huge potential with highly versatile opportunities however, they still bring great challenges before achieving successful clinical results in the treatment of AD. One for all, and the most controversial, is represented by the difficult passage through the naturally built BBB with therapeutic doses of drugs targeted at specific brain areas in a selective and safe manner (Saraiva et al., 2016; Zhang et al., 2018). What’s more, critical issues related to the biological safety and efficacy of nanomedicines before their clinical translation must be carefully taken into account by evaluating short- and long-term risks for human health. This, in turn, can be obtained by *in vitro* and *in silico* evidence of the molecular mechanisms underlying the effects of nano-based therapeutics before their clinical development for medical applications (Barz, 2015; Talekar et al., 2015). Approved and validated medical protocols also for the use of brain-targeted nanomedicines are still in their infancy and will represent the next necessary step, going through a decision-making process led by the relevant regulatory authorities. This step will be required to move forward from pre-clinical proof of concept to definitive therapeutic demonstration in a clinical setting. Despite this critical aspect, more nanoparticle-based therapeutics is expected to become available in the near future, with the goal to fulfill official guidelines approved by the international scientific and medical community (Hare et al., 2017; Hua and Wu, 2018; Cano et al., 2021).

What’s sure is that nanotechnologies are slowly revolutionizing medical strategies used to manage AD, hopefully pointing the way to help treat the disease, although there are still key bottlenecks to deal with, which we have discussed in the present review article.

## Author contributions

LLB wrote the manuscript and prepared the figures and tables. EM wrote the manuscript and prepared the figures. MDA supervised, revised, and critically discussed the manuscript. MG conceived the idea, wrote and supervised the manuscript, and prepared figures and tables. All authors contributed to the article and approved the submitted version.
